# Phylogenetic Group Distribution of Uropathogenic *Escherichia coli* and Related Antimicrobial Resistance Pattern: A Meta-Analysis and Systematic Review

**DOI:** 10.3389/fcimb.2022.790184

**Published:** 2022-02-25

**Authors:** Mehrdad Halaji, Amirhossein Fayyazi, Mehdi Rajabnia, Donya Zare, Abazar Pournajaf, Reza Ranjbar

**Affiliations:** ^1^ Infectious Diseases and Tropical Medicine Research Center, Health Research Institute, Babol University of Medical Sciences, Babol, Iran; ^2^ Department of Microbiology, School of Medicine, Babol University of Medical Sciences, Babol, Iran; ^3^ Department of Microbiology, School of Medicine, Shahid Beheshti University of Medical Sciences, Tehran, Iran; ^4^ Department of Microbiology, School of Medicine, Isfahan University of Medical Sciences, Isfahan, Iran; ^5^ Molecular Biology Research Center, Systems Biology and Poisonings Institute, Baqiyatallah University of Medical Sciences, Tehran, Iran

**Keywords:** uropathogenic *Escherichia coli*, polygenetic groups, antibiotic resistant, virulence factor, meta-analysis

## Abstract

The phylogenetic classification of *Escherichia coli* isolates is of great importance not only for understanding the populations of *E. coli* but also for clarifying the relationship between strains and diseases. The present study aimed to evaluate the prevalence of phylogenetic groups, antibiotic susceptibility pattern, and virulence genes among uropathogenic *E. coli* (UPEC) isolated from different parts of Iran through a systematic review and meta-analysis. Several international electronic sources, including Web of Science, PubMed, Scopus, and Embase, were searched (2000–2020) in order to identify the studies compatible with our inclusion criteria. The meta-analysis was performed using the metaprop program in the STATA (version 11) software. Based on our comprehensive search, 28 studies meeting the eligibility criteria were included in the meta-analysis. The pooled prevalence of phylogroups B_2_, D, B_1_, and A was 39%, 26%, 18%, and 8%, respectively. In addition, there was a significant heterogeneity among different phylogroups. However, according to the results of Begg’s and Egger’s tests, there were no significant publication bias in phylogroups B_2_, D, B_1_, and A. This research provided the first comprehensive study on phylogroups of UPEC isolated in Iran. Our findings indicated that phylogroup B_2_ and group D were the most predominant phylogenetic groups among UPEC isolates in various regions of Iran. In addition, we observed that certain phylogenetic groups are more antibiotic resistant than the others. It was also observed that the dissemination of virulent phylogroup B_2_ and D should be controlled *via* comprehensive infection control measures. Additionally, certain strategies should be developed for monitoring the antibiotic therapy.

## Introduction

Urinary tract infections (UTI), as one of the most prevalent pathological disorders, are the most problematic infectious diseases worldwide in both community and hospital settings (Terlizzi et al., 2017). UTI patients could be categorized into symptomatic and asymptomatic cases. Symptomatic UTI patients can be classified based on the degree of the severity into three classes, namely pyelonephritis (upper UTI, with kidney infection), cystitis (lower UTI, bladder infection), and urosepsis (Foxman, 2014; Smelov et al., 2016). Approximately 11 million individuals with UTI in the USA are annually referred to health centers, among whom 470,000 are hospitalized, incurring about $6 billion annually. It is known that Uropathogenic *Escherichia coli* (UPEC) is the main factor causing UTIs (Navidinia et al., 2018). The primary cause of UTIs is UPEC, both in community and hospital settings, which leads to a considerable rate of global mortality and morbidity (Tabasi et al., 2015). Antibiotic therapy is the only treatment for UTI; however, global spread of MDR bacterial strains has become a public health threat and major concern, particularly in patients with recurrent UTIs (Halaji et al., 2020; Sadeghi et al., 2020). Excessive use of a broad spectrum of antibiotics, such as fluoroquinolones, cephalosporins, and aminoglycosides, raise the cost of treatment and hospitalization (Fayyazi et al., 2020).

The major etiological agents associated with UTI are UPECs with the ability to successfully colonize the urinary tract. UPEC pathogenesis during UTIs occurs in an ascending manner as the following order: from the urethra to bladder to ureter to kidney to bloodstream (Terlizzi et al., 2017). A broad range of virulence factors and specific genes are expressed in UPEC isolates (Sadeghi et al., 2020). In fact, certain host cell types, including the stratified layers of bladder urothelium, such as differentiated superficial facet cells, less mature intermediate cells, and basal epithelial cells, can be breached by UPEC. Host cell invasion could facilitate the establishment and permanence of UPEC within the urinary tract (Lewis et al., 2016; Raeispour and Ranjbar, 2018).

In order to classify *E. coli* strains into one of the major phylogenetic classes of A, B_1_, B_2_, or D, a rapid and easy phylogenetic grouping technique based on triplex PCR has been developed to detect the genes *chuA*, *yjaA*, and *TspE4*. To improve the accuracy of their system, an extra gene target, *arpA*, has been added. This new quadruplex PCR is able to correctly assign *E. coli* strains in eight phylogroups, namely A, B_1_, B_2_, C, D, E, F, and one *Escherichia* cryptic clade I (Clermont et al., 2013; Najafi et al., 2018; Caméléna et al., 2019). The majority of strains responsible for extraintestinal infections belong to group B_2_ or to a lesser extent, to group D, while intestinal pathogenic and commensal isolates are observed in A and B_1_ (Molina-López et al., 2011; Lee et al., 2016). The phylogenetic classification of *E. coli* isolates is of great importance not only for understanding the populations of *E. coli* but also for clarifying the relationship between strains and diseases. *E. coli* sequence type 131 (ST131) is considered an important emerging pathogen among B_2_ strains, harboring multiple genes for resistance and virulence factors (VFs). The strains belonging to this group are mediated by the production of extended spectrum β-lactamases (ESBLs) and are resistant against most β-lactam antibiotics (Cristea et al., 2019). Expression of various markers of genetic virulence helps UPEC to cause infection within the urinary tract of the host (Picard et al., 1999; Johnson et al., 2001; Cristea et al., 2019). To the best of our knowledge, there is no available comprehensive information on the prevalence of phylogenetic groups and the related antibiotic susceptibility pattern and virulence genes among Iranian patients. Thus, the present study was conducted to determine the prevalence and distribution of phylogenetic groups, antibiotic susceptibility pattern, and virulence genes among UPEC isolated from different parts of Iran *via* a systematic review and meta-analysis.

## Material and Methods

### Search Strategies

The current study was carried out according to the Preferred Reporting Items for Systematic Reviews and Meta-Analyses (PRISMA) guidelines (**Supplementary Data**). A systematic literature search was conducted in the Web of Science, PubMed, Scopus, and Embase electronic databases. The search was limited to the articles published by Iranian authors from the beginning to the end of November 2020. The following terms, “*Escherichia coli*” OR “*E. coli*” OR “UPEC” OR “uropathogenic *E. coli*” OR “uropathogenic *Escherichia coli*” AND “phylogenetic group” OR “phylogroups” OR “phylogroup” OR “phylotypes “OR “phylogroups” AND “IRAN,” were searched as scientific keywords and phrases in the present survey.

### Inclusion and Exclusion Criteria

To determine the articles meeting the inclusion criteria and reduce the risk of error, two reviewers screened independently the databases with the related keywords and reviewed the titles, abstracts, and full texts, and any discrepancies were resolved by consensus. The articles with the following criteria were included in the study: (1) cross-sectional, retrospective, and cohort studies indexed in the Web of Science or PubMed or Scopus database and reporting the prevalence of phylogenetic groups in *E. coli* isolates collected from the urine of patients with UTI and (2) those published worldwide with available English abstracts. Review articles, meta-analysis, or systematic articles, editorials, case report studies, letters to the editors, congress and meeting abstracts, studies where the sample size contains less than 10 isolates, studies with samples from environmental or nonclinical sources, articles without full text, duplicate publications, and articles with unclear and missing data were excluded.

### Quality Assessment and Data Extraction

Five eligibility and quality assessment criteria were retrieved based on the Joanna Briggs Institute guidelines, and any disagreements were resolved by consensus. The following data were extracted for eligible studies: authors’ names, publication year, performed time, study location, characterization of the studied population, sample size, prevalence of phylogroups, virulence factor, and antibiotic resistance pattern.

### Statistical Analysis

Analysis of data was carried out using the metaprop program in STATA statistical software, version 11.0 (Stata, College Station, TX, USA) (Nyaga et al., 2014). The pooled prevalence of phylogenic groups and associated antibiotic resistance and virulence factor with 95% confidence intervals (95% CIs) were estimated through the random effects model. In this meta-analysis, the CIs for proportions were computed using the score method. Statistical heterogeneity between the studies was calculated utilizing the Cochran Q Chi-square test and Cochrane *I* ^2^. The funnel plot, Begg’s rank correlation test, and Egger’s weighted regression tests were conducted to evaluate possible publication bias and any asymmetry appearing in the funnel plot, or *p* < 0.05 in the test was indicative of statistically significant publication bias (Begg, 1985). Possible sources of heterogeneity were calculated employing meta-regression analysis, and the subgroup analysis was performed based on the location of the study (region) and the types of patients (Zeng et al., 2015). Moreover, the sensitivity analysis was assessed with influence analysis and ignoring each study, followed by evaluating the estimated pooled prevalence in the absence of the excluded studies. In addition, the confounding effect of possible confounders, such as the time of the study (performed years), was evaluated by conducting meta-regression analyses.

## Results

### Database Search and Characterization of Studies

**Figure 1:**
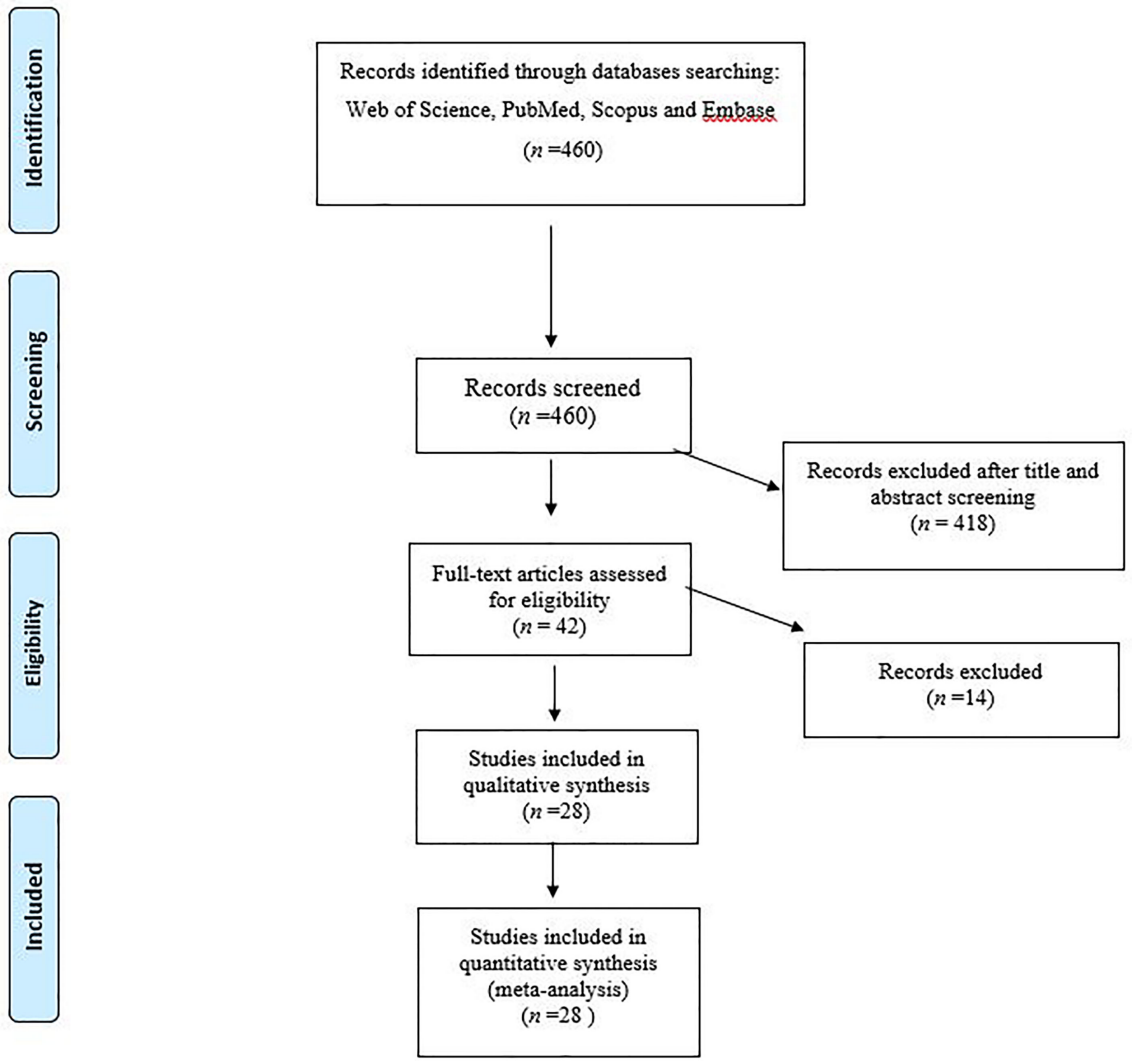
Flow chart of the study selection for inclusion in the systematic review.

Based on our comprehensive search, 28 studies with eligibility criteria (**Figure 1**) were included in the meta-analysis (Ramos et al., 2011; Navidinia et al., 2013; Adib et al., 2014; Alizade et al., 2014a; Alizade et al., 2014b; Hemati et al., 2014; Kazemnia et al., 2014; Derakhshandeh et al., 2015; Iranpour et al., 2015; Rahdar et al., 2015; Salmani et al., 2016; Sohrabi and Zeighami, 2016; Hashemizadeh et al., 2017; Hojabri et al., 2017; Salehzadeh and Zamani, 2018; Bahadori et al., 2019; Farajzadah Sheikh et al., 2019; Norouzian et al., 2019; Shahin et al., 2019; Staji et al., 2019; Bakhtiari et al., 2020; Moez et al., 2020; Ranjbar et al., 2020; Yazdanpour et al., 2020). Of the 28 included studies, 17 and 9 studies reported the prevalence of phylogenic groups from hospitalized and community patients, respectively. Also, two analyses were performed on both groups of patients. The full characteristics of the included studies are listed in **Table 1**.

**Table 1:** The main characteristics of studies included in the meta-analysis.

Author	Published time	City	Region	Population studies	Number of *E. coli* isolates	A	B_1_	B_2_	D	ST131	References
**Ramos et al.**	2011	Tehran	North	Hospitalized	48	–	–	21	–	–	Ramos et al. (2011)
**Navidinia et al.**	2013	Tehran	North	Hospitalized	50	4	2	27	17	–	Navidinia et al. (2013)
**Adib et al.**	2014	Kerman	Southeast	Hospitalized	137	63	18	27	29	–	Adib et al. (2014)
**Alizade et al.**	2014	Kerman	Southeast	Hospitalized	45	4	7	19	15	–	Alizade et al. (2014a)
**Kazemnia et al.**	2014	Urmia	North of West	Hospitalized	25	8	0	10	7	–	Kazemnia et al. (2014)
**Alizadeh et al.**	2014	Kerman	Southeast	Community	122	55	5	9	53	–	Alizade et al. (2014b)
**Hemmati et al.**	2014	Kerman	Southeast	Community	92	23	8	29	32	–	Hemati et al. (2014)
**Iranpour and Najafi et al.**	2015	Bushehr	South	Hospitalized	140	1	7	55	4	–	Iranpour et al. (2015)
**Derakhshandeh et al.**	2015	Shiraz	South	Hospitalized	85	56	–	15	14	–	Derakhshandeh et al. (2015)
**Rahdar et al.**	2015	Zabol	Southeast	Community	100	17	6	55	22	–	Rahdar et al. (2015)
**Sohrabi et al.**	2016	Zanjan	West	Hospitalized	137	16	–	92	29	–	Sohrabi and Zeighami (2016)
**Salmani et al.**	2016	Tehran Sanandaj	–	Hospitalized	104	32	26	26	20	–	Salmani et al. (2016)
**Hashemizadeh et al.**	2017	Kerman	Southeast	Hospitalized and community	251	35	31	99	86	–	Hashemizadeh et al. (2017)
**Hashemizadeh et al.**	2017	Kerman	Southeast	Hospitalized	100	21	15	34	30	4 (B_2)_	Hashemizadeh et al. (2017)
										2 (A)	
**Hashemizadeh et al.**	2017	Kerman	Southeast	Community	151	14	16	65	56	–	Hashemizadeh et al. (2017)
**Salehzadeh et al.**	2017	Rasht	North	Community	100	14	6	52	28	–	Salehzadeh and Zamani (2018)
**Hojabri et al.**	2017	Semnan	North	Hospitalized	339	–	–	136	33	62 (B_2_)	Hojabri et al. (2017)
										1 (F)	
**Staji et al.**	2019	Semnan	North	Community	160	25	12	75	48	–	Staji et al. (2019)
**Yazdanpour et al.**	2020	Zabol	Southeast	Community	248	16	12	167	53	–	Yazdanpour et al. (2020)
**Farajzadah Sheikh et al.**	2019	Ahvaz	Southwest	Hospitalized and community	232	37	18	42	135	–	Farajzadah Sheikh et al. (2019)
**Farajzadah Sheikh et al.**	2019	Ahvaz	Southwest	Hospitalized	139	19	9	23	90	–	Farajzadah Sheikh et al. (2019)
**Farajzadah Sheikh et al.**	2019	Ahvaz	Southwest	Community	93	18	9	19	45	–	Farajzadah Sheikh et al. (2019)
**Bahadori et al.**	2018	Shiraz	South	Community	90	13	6	53	18	–	Bahadori et al. (2019)
**Norouzian et al.**	2019	Tehran	North	Hospitalized	106	22	10	52	22	–	Norouzian et al. (2019)
**Najar Peerayeh et al.**	2019	Tehran	North	Hospitalized	16	3	2	7	4	–	Shahin et al. (2019)
**Morovati et al.**	2020	Hamadan	West	Hospitalized	140	23	8	39	4	–	Moez et al. (2020)
**Ranjbar et al.**	2020	Tehran	North	Hospitalized	60	1	3	50	6	–	Ranjbar et al. (2020)
**Bakhtiari et al.**	2020	Hamadan	West	Hospitalized	113	23	5	50	35	–	Bakhtiari et al. (2020)

### Prevalence of Phylogroup B_2_

**Figure 2:**
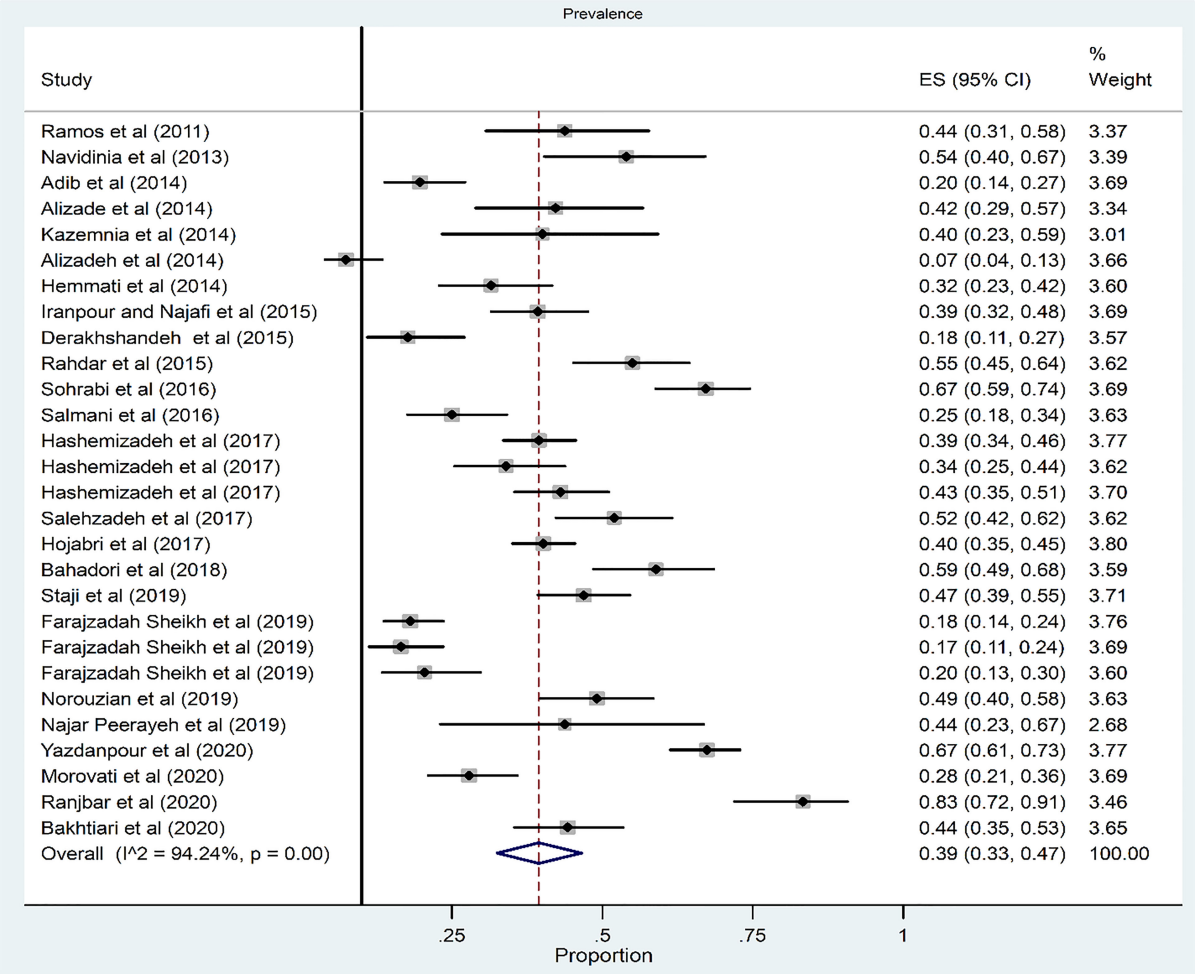
Forest plot of the meta-analysis of phylogroup B_2_ prevalence among UPEC isolates.

**Figure 3:**
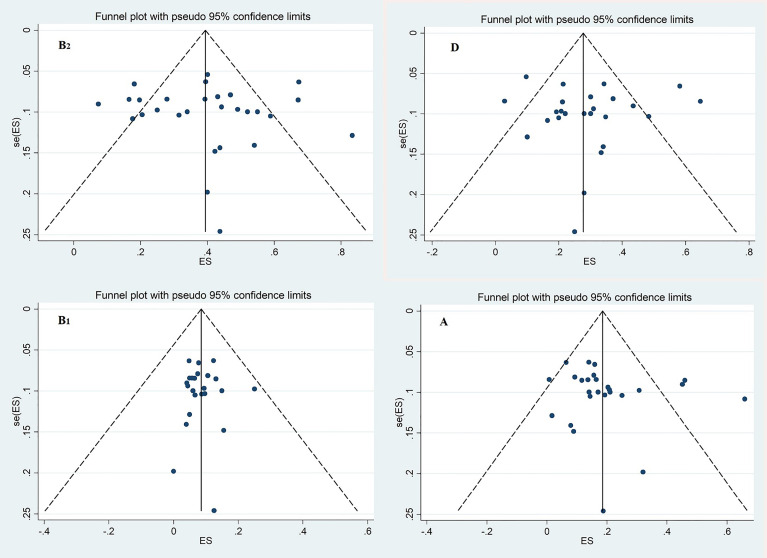
Funnel plot for evaluation of publication bias; Phylogenetic groups B_2_, D, B_1_ and A.

The pooled prevalence of phylogroup B_2_ among 28 studies was 39% (95% CI: 33–47) (**Figure 2**). There was a significant heterogeneity among the 28 studies (*χ* ^2^ = 468.47; *p* < 0.001; *I* ^2^ = 94.24%). The funnel plot for publication bias did not show any evidence of asymmetry (**Figure 3**). According to the results of Begg’s (*Z* = 0.51, *p* = 0.60) and Egger’s tests (*t* = 0.27, *p* = 0.79), there was no significant publication bias (**Figure 3**).

The subgroup analysis results based on region indicated that the highest and lowest overall occurrence of phylogroup B_2_ was 52% and 18% in the north and southwest regions, respectively (**Supplementary Figure 1** and **Table 1**). The subgroup analysis results based on source of patients indicated that the overall prevalence of phylogroup B_2_ in the hospitalized and community patients was 40% and 41%, respectively (**Supplementary Figure 2** and **Table 1**).

### Prevalence of Phylogroup D

**Figure 4:**
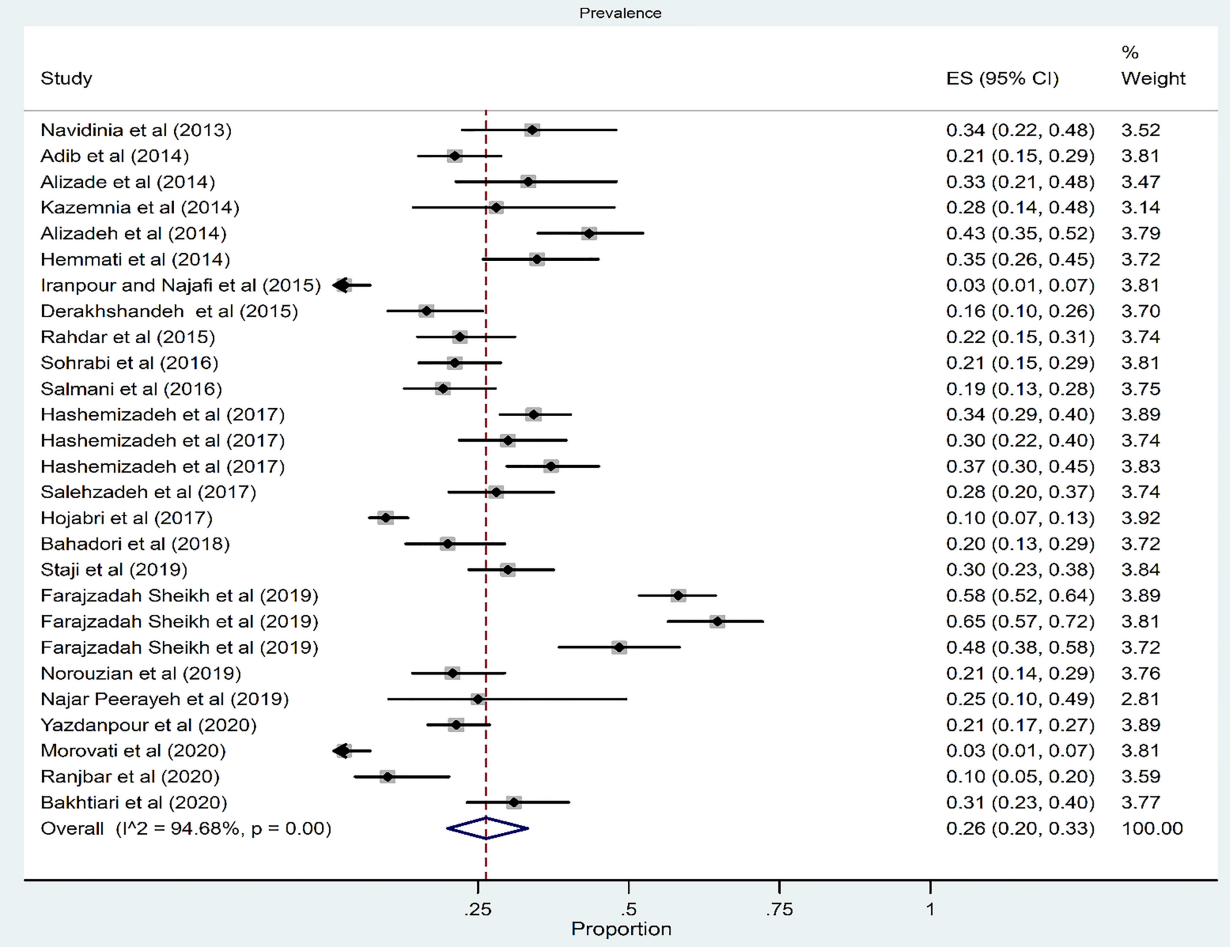
Forest plot of the meta-analysis of phylogroup D prevalence among UPEC isolates.

Among 27 studies, the pooled prevalence of phylogroups D was 26% (95% CI: 20–33) (**Figure 4**). There was a significant heterogeneity for phylogroup D among the 27 studies (*χ* ^2^ = 488.47; *p* < 0.001; *I* ^2^ = 94.68%). The funnel plot for publication bias in the four phylogroups did not show any evidence of asymmetry (**Figure 3**). Accordingly, the results of Begg’s (*Z* = 0.1, *p* = 0.91) and Egger’s tests (*t* = 0.09, *p* = 0.93) showed no significant publication bias in phylogroup D (**Figure 3**).

The subgroup analysis results based on region indicated that the highest and lowest overall prevalence of phylogroup D was in the southwest (58%) and south (12%) regions, respectively (**Supplementary Figure 3** and **Table 2**).

The subgroup analysis results based on source of patients indicated that the overall prevalence of phylogroup D was 21% in hospitalized and 31% in community patients (**Supplementary Figure 4** and **Table 2**).

### Prevalence of Phylogroup A

**Figure 5:**
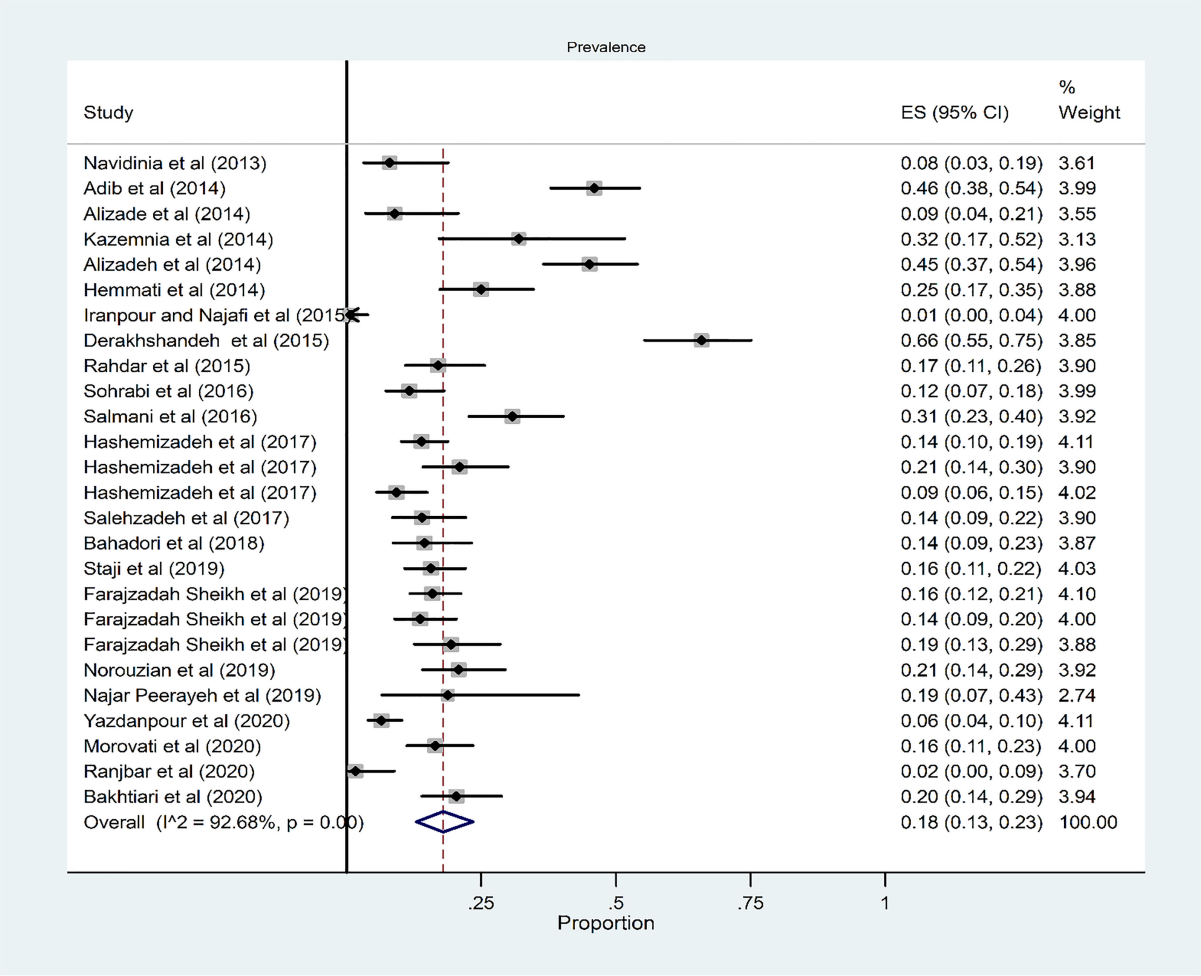
Forest plot of the meta-analysis of phylogroup A prevalence among UPEC isolates.

The pooled prevalence of phylogroup A among 26 studies was 18% (95% CI: 13–23) (**Figure 5**). There was a significant heterogeneity among the 26 studies (*χ* ^2^ = 341.53; *p* < 0.001; *I* ^2^ = 92.68%). The funnel plot for publication bias in the four phylogroups did not show any evidence of asymmetry (**Figure 3**). According to the results of Begg’s (*Z* = 1.52, *p* = 0.36) and Egger’s tests (*t* = 0.93, *p* = 0.36), there was no significant publication bias (**Figure 3**). The subgroup analysis results based on region indicated that the highest and lowest overall occurrence of group A was in the northwest (32%) and north (12%) regions, respectively (**Supplementary Figure 5** and **Table 3**). The subgroup analysis results based on source of patients indicated that the overall prevalence of phylogroup A in the hospitalized and community patients were 19% and 17%, respectively (**Supplementary Figure 6** and **Table 3**).

### Prevalence of Phylogroup B_1_

**Figure 6:**
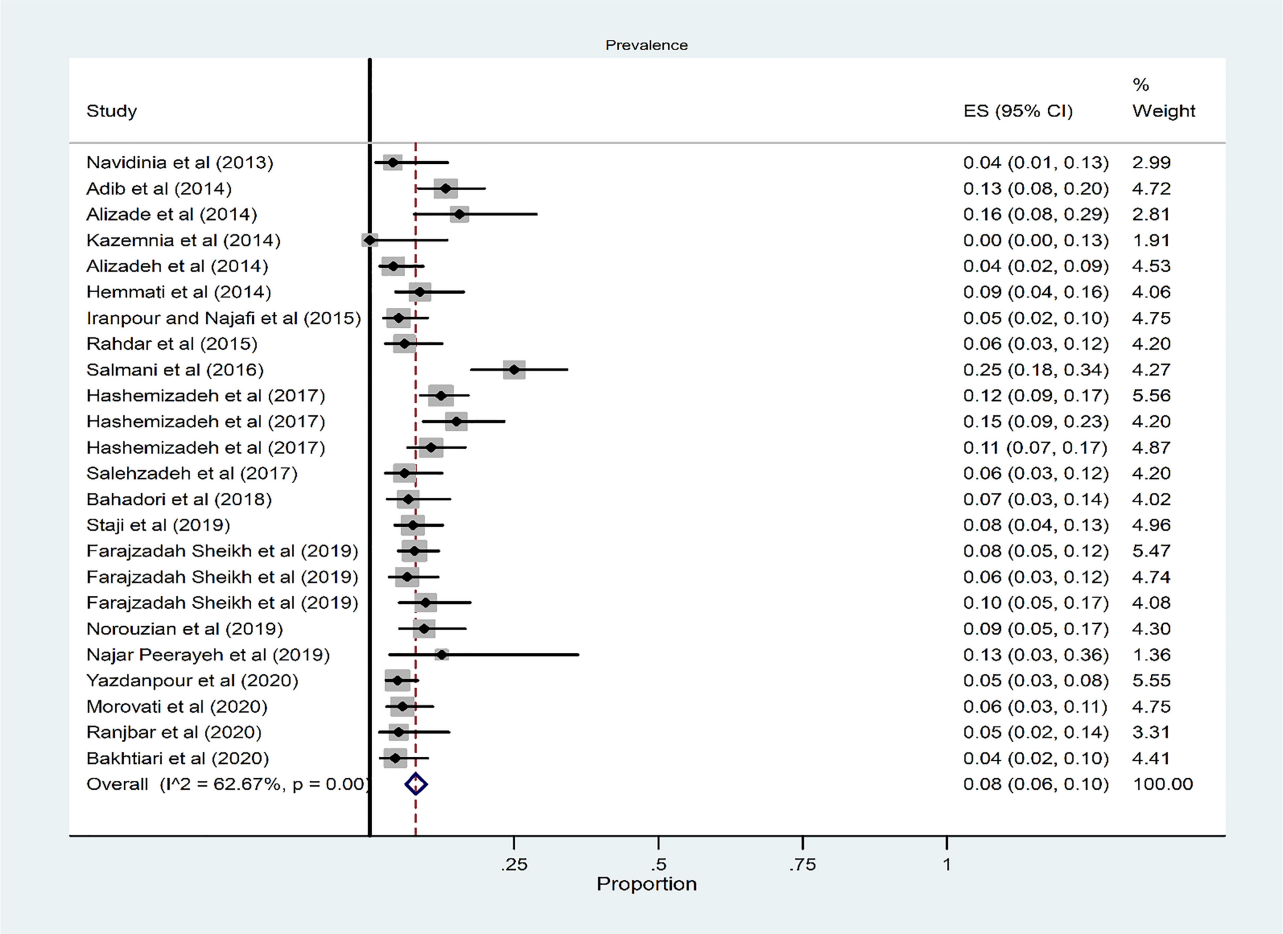
Forest plot of the meta-analysis of phylogroup B_1_ prevalence among UPEC isolates.

From 24 studies, the pooled prevalence of phylogroup B_1_ was 8% (95% CI: 6–10) (**Figure 6**). There was a significant heterogeneity for phylogroup B_1_ among the 24 studies (*χ* ^2^ = 61.62; *p* < 0.001; *I* ^2^ = 62.67%). According to the results of Begg’s (*Z* = 0.07, *p* = 0.94) and Egger’s tests (*t* = 0.06, *p* = 0.95) for phylogroup B_1_, there was no significant publication bias (**Figure 3**).

The subgroup analysis results based on region indicated that the highest and lowest overall occurrence of phylogroup B1 was 10% in the west and 0% in the south region (**Supplementary Figure 7** and **Table 4**). Also, the overall prevalence of phylogroup B_1_ was 8% and 7% in the hospitalized and community patients, respectively (**Supplementary Figure 8** and **Table 4**).

### Sensitivity Analysis and Meta-Regression

**Figure 7:**
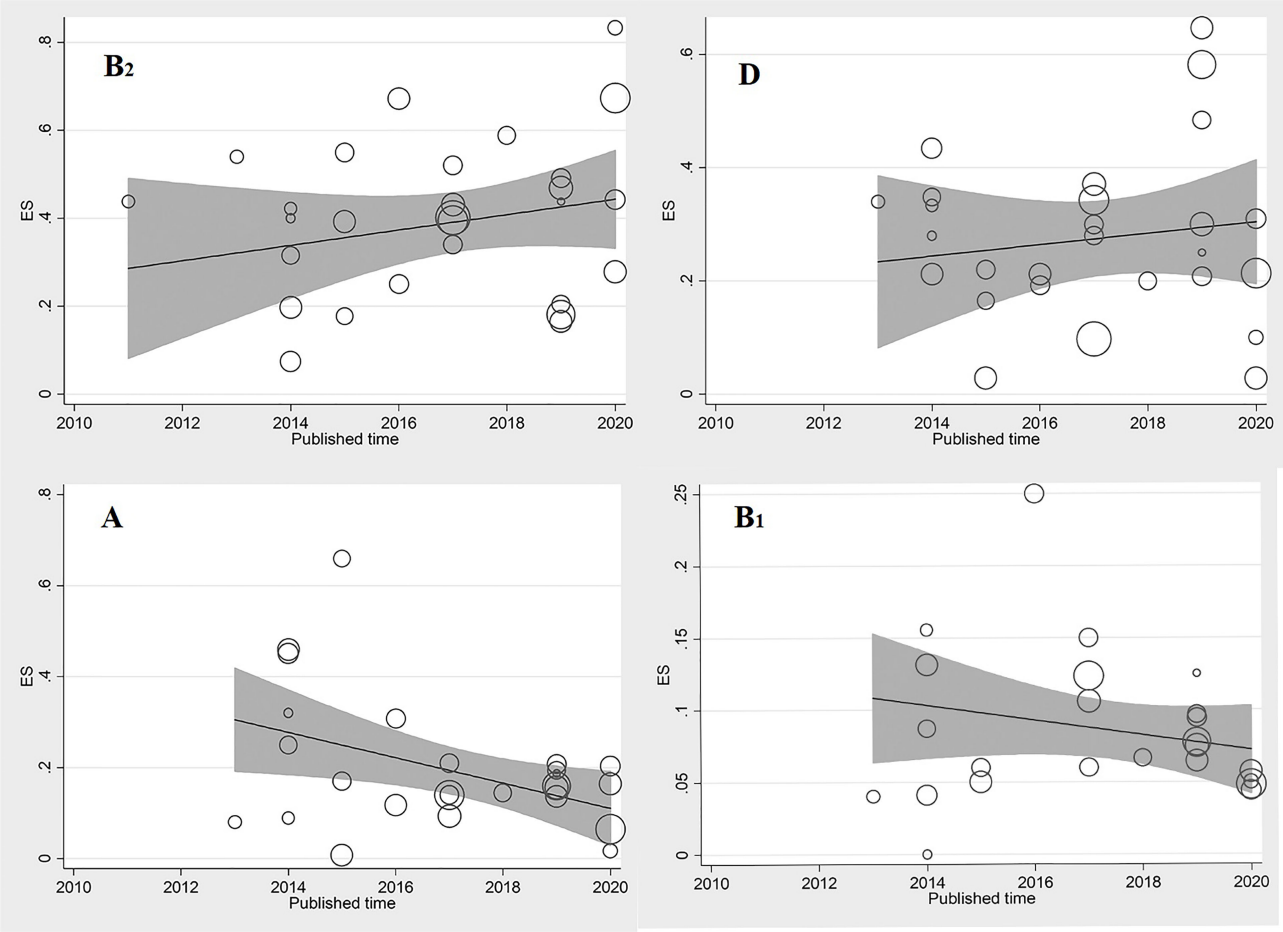
Meta regression of the log-event rates by year (phylogroups B_2,_ D, A, and B_1_).

Meta-regression results indicated that the prevalence of phylogroups B_2_, D, and B_1_ among UPEC isolates was not significantly associated with year, coefficients: 0.01442 (95% CI: −0.01533–0.04418, *p* = 0.32), 0.005076 (95% CI: −0.025178–0.03533, *p* = 0.73), and −0.00513 (95% CI: −0.02311–0.01283, *p* = 0.55), respectively. Furthermore, no significant increasing trend was observed over time on the estimated pooled prevalence of phylogroups B_2_, D, and B_1_ in the included studies (**Figure 7**).

Meta-regression results indicated that the prevalence of phylogroup A among UPEC isolates was significantly associated with year, coefficients: 0.026443 (95% CI: −0.05079–0.00209, *p* = 0.034). Furthermore, significant decreasing trend was observed over time on the estimated pooled prevalence of phylogroup A in the included studies (**Figure 7**).

Moreover, the results of the influence analysis demonstrating that none of the studies affect the estimated pooled prevalence of phylogroups B_2_, D, A, and B_1_. In addition, we excluded each study and examined the outcome. The sensitivity analyses also showed no significant change in the estimated pooled prevalence in the meta-analysis results after excluding studies with relatively lower quality [**Supplementary Figure 9** (A: B_2_, B: D, C: A, D: B_1_)].

### Prevalence of Antibiotic Resistance Among Phylogroups

According to the antibiotic resistance pattern in phylogroup B_2_, ampicillin had the highest antibiotic resistance rate at 76% (95% CI: 58–90; *I* ^2^ = 89.57%; *n* = 6 studies), followed by cefixime at 70% (95% CI: 34–96; *I* ^2^ = 90.84%; *n* = 3 studies), while nitrofurantoin and imipenem had the lowest resistance rates at 2% (95% CI: 0–5; *I* ^2^ = 35.65%; *n* = 5 studies) and 7% (95% CI: 0–21; *I* ^2^ = 92.52%; *n* = 6 studies), respectively (**Supplementary Table 5**).

In phylogroup D, the highest antibiotic resistance rate was against cefixime with 80% (95% CI: 53–98; *I* ^2^ = 45.54%; *n* = 3 studies), followed by ampicillin with 64% (95% CI: 28–94; *I* ^2^ = 93.87%; *n* = 6 studies), while amikacin 5% (95% CI: 0–15; *I* ^2^ = 60.68%; *n* = 5 studies) and ceftriaxone 17% (95% CI: 0–54; *I* ^2^ = 78.02%; *n* = 4 studies) had the lowest antibiotic resistance rate. Also, in this group, there was no resistance to nitrofurantoin (**Supplementary Table 6**).

In group A, ampicillin had the highest antibiotic resistance rate at 59% (95% CI: 27–89; *I* ^2^ = 75.65%; *n* = 6 studies), followed by nalidixic acid at 58% (95% CI: 6–100; *I* ^2^ = 93.45%; *n* = 6 studies), while imipenem and amikacin were the most effective antibiotics with resistance rates of 1% (95% CI: 0–12; *I* ^2^ = 61.83%; *n* = 6 studies) and 3% (95% CI: 0–16; *I* ^2^ = 58.77%; *n* = 5 studies), respectively. Also, there was no resistance to nitrofurantoin in this group (**Supplementary Table 7**).

Moreover, the highest antibiotic resistance rate in group B_1_ was against ampicillin with 32% (95% CI: 0–90; *I* ^2^ = 3.01%; *n* = 6 studies), followed by ciprofloxacin with 30% (95% CI: 16–46; *I* ^2^ = 0%; *n* = 5 studies), while nalidixic acid 1% (95% CI: 0–56; *I* ^2^ = 53.96%; *n* = 6 studies) and gentamicin 2% (95% CI: 0–10; *I* ^2^ = 27.07%; *n* = 6 studies) had the lowest antibiotic resistance rates. Also, in this group, there was no resistance to imipenem, cefotaxime, amikacin, nitrofurantoin, and ceftazidime (**Supplementary Table 8**).

### Prevalence of Virulence Factor Among Phylogroups

The results of the investigation of virulence factors into phylogenetic groups indicated that the highest prevalence among all groups was related to gene *fimH*, followed by gene *papC.*

In group B_2_, *afa* and *hly* genes with 7% and 21% frequency had the lowest prevalence among virulence factors (**Supplementary Table 9**). In group D, the lowest prevalence of virulence factors was related to gene *hly* with 10% and *papEF* with 11% (**Supplementary Table 10**). Also, in group A, the lowest prevalence of virulence factors was related to gene *afa* with 1% and *cnfi* with 1% (**Supplementary Table 11**). Finally, *papEF* and *hly* genes with 4% and 5% prevalence were the lowest prevalence in group B_1_ (**Supplementary Table 12**).

### Prevalence of ST131 Among Phylogroups

Our finding revealed that among all of the studies included, only two reported ST131 among different phylogenetic groups. In total, sixty-nine isolates were ST131; of these isolates, 66, 2, and 1 belong to phylogenetic groups B_2_, A, and F, respectively.

## Discussion

According to the phylogenetic background, *E. coli* strains showed moderate levels of recombination in the species (Tenaillon et al., 2010; Dixit et al., 2015; Touchon et al., 2020) and a strong phylogenetic structure with eight main phylogroups, four of which (A, B1, B2, and D) showed the majority of the strains and four others (C, E, F, and G) are more scarce. Moreover, these phylogroups apparently differ in their phenotypic and genotypic characteristics within and across phylogroups, such as their antibiotic-resistance profiles and their growth rate (Touchon et al., 2020).

Monitoring and evaluating the *E. coli* genotypic characteristics from urine resources provide useful data on the epidemiology of diseases in various geographical areas (Momtaz et al., 2013; Ranjbar and Farahani, 2018). To the best of our knowledge, the present work is the first comprehensive meta-analysis investigating the occurrence of phylogroup classes in UPEC isolates. We also investigated into the occurrence of phylogroup in different geographical locations and based on the type of patients.

Phylogenetic analysis indicated that the majority of UPEC isolates belonged to phylogroup B_2_ (39%), followed by group D (26%), group A, and group B_1_, which is in agreement with the study conducted by Munkhdelger et al. (2017), where B_2_ (33.8%) was the dominant phylogroup followed by D (28.4%), A (19.6%), and B_1_ (18.2%).

The majority of the studies on the phylogenetic grouping among UPEC have reported a similar distribution, such as studies conducted in China (Zhao et al., 2015), South Korea (Lee et al., 2016), Denmark (Ejrnæs et al., 2011), Pakistan (Bashir et al., 2012), Ethiopia (Dadi et al., 2020), Mexico (Paniagua-Contreras et al., 2017), and France (Dubois et al., 2010), in which it was found that the majority of isolates of *E. coli* predominantly belong to phylogenetic group B_2_. In this systematic review, we noted a high prevalence of phylogroup B_2_ in UPEC isolates, reflecting the importance of investigating and addressing the prevalence of isolates belonging to this group since they revealed a partially high level of antibiotic resistance and virulence factors.

Commensal populations of *E. coli* include stable genetic isolates with far lower recombination rates, resulting in a clonal population structure and allowing characterization of the main phylogenetic groups (Stoppe et al., 2017). Several previous studies have reported that phylogenetic groups A and B_1_ are mostly commensal *E. coli* isolates. In this regard, Duriez et al. (2001) and Khairy et al. (2019) reported that phylogenetic groups A comprised the highest proportion of phylogenetic groups among human commensal *E. coli* and UPEC strains.

Certain papers have revealed that phylogroup A was the leading phylogroup in UPEC isolates (Grude et al., 2007; Romanus and Eze, 2011; Derakhshandeh et al., 2015; Khairy et al., 2019). Our study on the other hand implied that 18% of the isolates belonged to phylogenetic group A, which was greater than that in the studies performed in South Korea (3.44%) (Lee et al., 2016). Such observations indicated that we should consider all the potential risks of phylogenetic group A compared with those in phylogroup B_2_ (Marialouis and Santhanam, 2016). Moreover, the predominance of the phylogenetic group A in UPEC isolates, which is normally related to the commensal strains, implies that the gastrointestinal tract is the main origin of strains colonizing the urinary tracts (Moreno et al., 2006; Khairy et al., 2019).

Additionally, phylogroup D has been found to be the leading strain in certain studies (Themphachana et al., 2015; Gao et al., 2017); this reveals that the colon may be the main reservoir for strains causing UTI. Nevertheless, the second most prevalent group has been reported to be phylogenetic group D among drug-resistant UPEC strains, exhibiting slighter phylogenetic shift towards group B_2_ (Johnson et al., 2003; Adwan et al., 2015).

These variations in the occurrence of the phylogenetic groups may be on account of host genetic factors, site of infection, geographical distribution, or variations in methodology, the origin of isolates, and differences in the sample size. Additionally, these factors may be considered to be a source of heterogeneity. The present systematic review illustrated a significant heterogeneity among different phylogroups in the 28 studies conducted in Iran. We performed subgroup analysis of certain factors that confound the assessment (types of patients and geographical distribution based on region) and meta-regression for controlling this heterogeneity.

The geographical distribution of phylogenetic groups is variable in different regions of Iran. Accordingly, the subgroup analysis of the geographical distribution indicated that the predominant prevalence of phylogroups B_2_, D, A, and B_1_ had a frequency of 52%, 58%, 32%, and 10% in the north, southwest, northwest, and west of Iran, respectively.

Moreover, based on our findings, the high prevalence of phylogroup D was found among the isolates obtained from hospital-acquired infection rather than community-acquired infection. However, several reports have indicated a discrepancy in the prevalence of these phylogroups in both community and hospital infections. Meanwhile, our results revealed no significant differences on pooled prevalence of phylogroups neither in community-acquired nor hospital-acquired infections.

According to meta-regression, it seems as though the trend of phylogroup B_2_ incidence increased gradually from 20% in 2014 up to 83% in 2020. This increasing trend seems to be directly linked to the increased UPEC infections in phylogroup B_2_.

Investigations have shown that we have been experiencing an increase in the frequency of members of virulent phylogroups from clinical samples since 2009 to date.

Once rank correlation approaches show bias, there are possibly minor study effects. Meanwhile, according to the meta-regression analysis, the weight of the studies should not be regarded as a confounding factor. In addition, based on the sensitivity analysis, the exclusion of any works does not have any considerable impacts on the approximated pooled prevalence.

Furthermore, a superior characteristic is presented by these phylogenetic groups due to their partially higher content of virulence factors, which makes them virulent clinical isolates and harder to treat. In our study, the occurrence of VFs was greater in within-group B_2_ isolates taken from the patients’ urine compared with the other phylogenetic groups. This is consistent with former studies performed in South Korea (Lee et al., 2016), Denmark (Ejrnæs et al., 2011), Pakistan (Bashir et al., 2012), Ethiopia (Dadi et al., 2020), Mexico (Paniagua-Contreras et al., 2017), and Poland (Kot et al., 2016).

Based on the VF distribution in the phylogenetic groups, the existence of some genes had a high incidence in groups B_2_, D, B_1_, and A compared with that in the other VFs, including *fimH papC* and *iucD*. In this regard, Karami et al. (2017) reported that *iutA* and *papC* are encoded on mobile elements or pathogenicity islands in uropathogenic strains.

In this regard, some reports conducted in Iran revealed that the most frequent PAI marker belonged to PAIIV536. This PAI marker contains iron uptake system encoding genes and appears to be vital for successful colonization and wellness of UPEC strains throughout the urinary tract. However, previous studies have reported that there are a lot of differences concerning PAI markers among phylogenetic groups (Samei et al., 2016; Najafi et al., 2018). Accordingly, Najafi et al. (2018) reported that the majority of the isolates belonging to phylogenetic group B_2_ had all the investigated PAI markers. Moreover, their findings revealed that B_1_, A, and D groups of UPEC isolates had fewer PAI markers (Najafi et al., 2018).

Therefore, the performance of virulence genes in group B_2_ strains is mostly over the pathogenicity islands in the chromosome; however, virulence genes are often carried by group D strains, such as *iutA*, on plasmids. Nevertheless, group B_2_ is highly genetically diverse with at least nine subgroups (Le Gall et al., 2007), some of which may act well in the incorporation of genetic elements transferred horizontally compared with the others.

The differences concerning the host characteristics, geographical differences, and strain types are therefore responsible for variations in distributing such virulence factors in isolated UPEC. This result could be justified by the fact that the *E. coli* strain related to phylogroup B_2_ comprises a partially higher number of virulence genes compared with the *E. coli* related to the other phylogroups in other works on UPEC isolates.

In our work, group B_2_ isolates had high levels of resistance against ampicillin, cefepime, nalidixic acid, and ceftazidime and less resistance against nitrofurantoin and imipenem; meanwhile, a member of group D had a high resistance level against cefepime and ampicillin and less resistance against nitrofurantoin and amikacin. This finding is consistent with that of Bashir et al. (2012) and on the contrary to that of Iranpour et al. (2015) (Iran) who found a low drug resistance level for group D isolates.

Moreover, regarding antibiotic resistance, high levels of resistance was observed in our work among the members of phylogroups D, followed by B_2_, A, and B_1._

Phylogenetic group D source was a considerable independent cause of antibiotic resistance, consistent with former studies, indicating that resistant genes could be achieved by isolates belonging to phylogenetic D.

There were certain limitations in our systematic review; primarily, phylogroups of UPEC have not yet been examined in numerous areas of Iran. Therefore, the frequency of phylogroups could not be completely represented. Furthermore, considering the heterogeneity found within the considered studies, the findings should be cautiously interpreted.

## Conclusion

The results of the present study provided beneficial epidemiological information about the distribution of phylogroups in UPEC from Iranian patients. Our findings shed light on the fact that phylogroup B_2_ and group D were the most predominant phylogenetic groups among UPEC isolates in various regions of Iran, which is comparable with other parts of the world. Due to the relatively high frequency of phylogroup B_2_ and group D strains, it is necessary to pay attention to various groups involved in clinical care. Moreover, our results suggested that the members of phylogroup B_2_ strains may become reservoirs of genes encoding virulence factors. In addition, certain polygenetic groups were found to be more resistant than the others, which could be due to greater exposure of certain phylogenetic groups to antimicrobial agents. The dissemination of virulent phylogroups B_2_ and D could be suggested to be controlled through comprehensive infection control measures and through developing strategies for monitoring antibiotic therapy.

## Author Contributions

Conceived and designed the experiments: RR and MH. Performed the experiments: MH, AF, and DZ Analyzed the data: AF and DZ. Contributed reagents/materials/analysis tools: MR and MH. Contributed to the writing of the manuscript: RR, AF, and MH. Manuscript revision and English editing: AP and MR. All authors read and approved the final manuscript.

## Funding

This study was financially funded by the Babol University of Medical Sciences [Grant no. 724133882].

## Conflict of Interest

The authors declare that the research was conducted in the absence of any commercial or financial relationships that could be construed as a potential conflict of interest.

## Publisher’s Note

All claims expressed in this article are solely those of the authors and do not necessarily represent those of their affiliated organizations, or those of the publisher, the editors and the reviewers. Any product that may be evaluated in this article, or claim that may be made by its manufacturer, is not guaranteed or endorsed by the publisher.
