# Supplementary material for: Phylogenetic Group Distribution of Uropathogenic Escherichia coli and Related Antimicrobial Resistance Pattern: A Meta-Analysis and Systematic Review
Source: Front Cell Infect Microbiol. 2022 Feb 25;12:790184. doi: 10.3389/fcimb.2022.790184 (PMC8914322; doi:10.3389/fcimb.2022.790184)
Supplement: Supplementary file 1 [file DataSheet_1.docx]

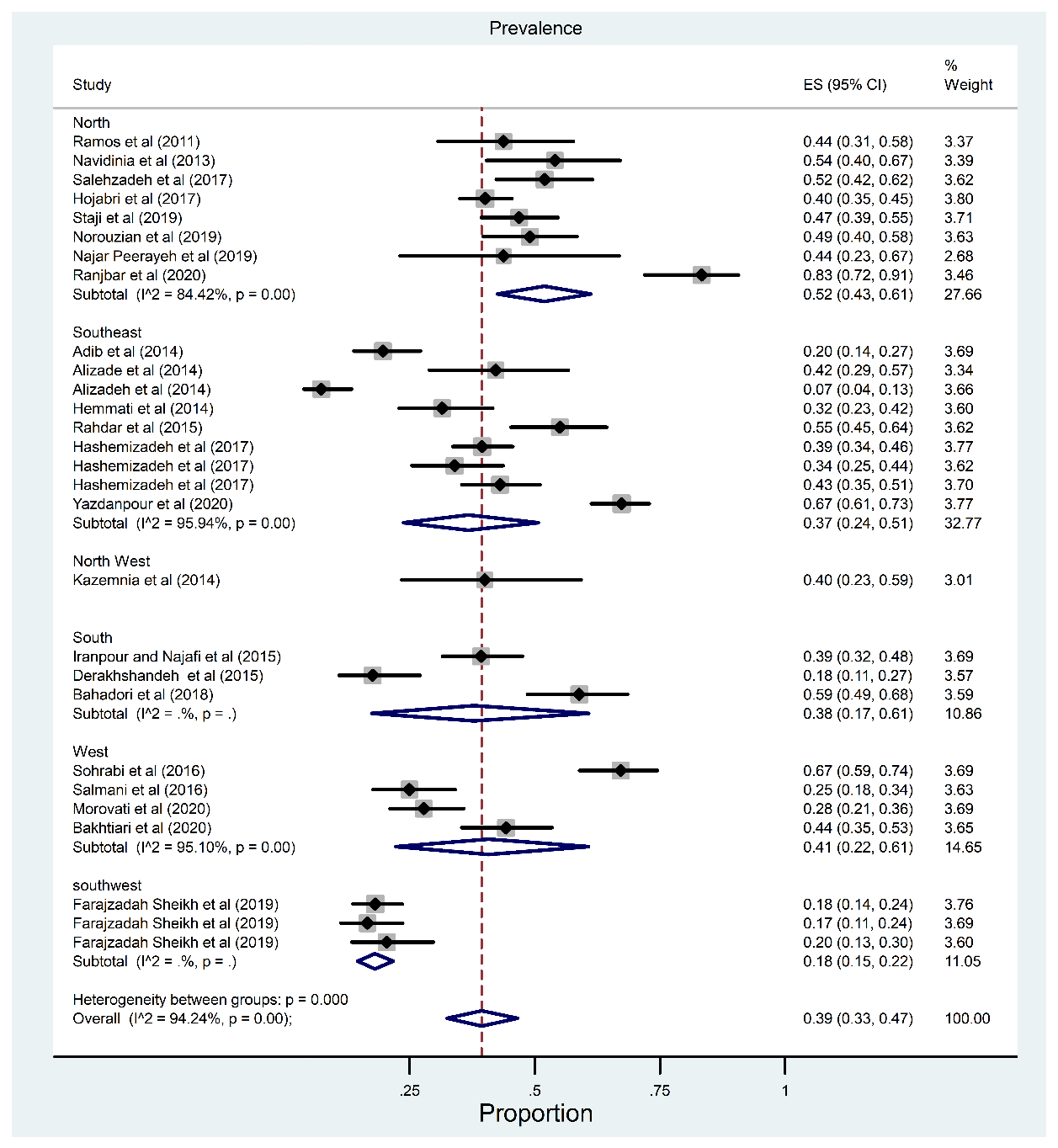


Figure 1: The subgroup analysis results based on region (Phylogroup B_2_)


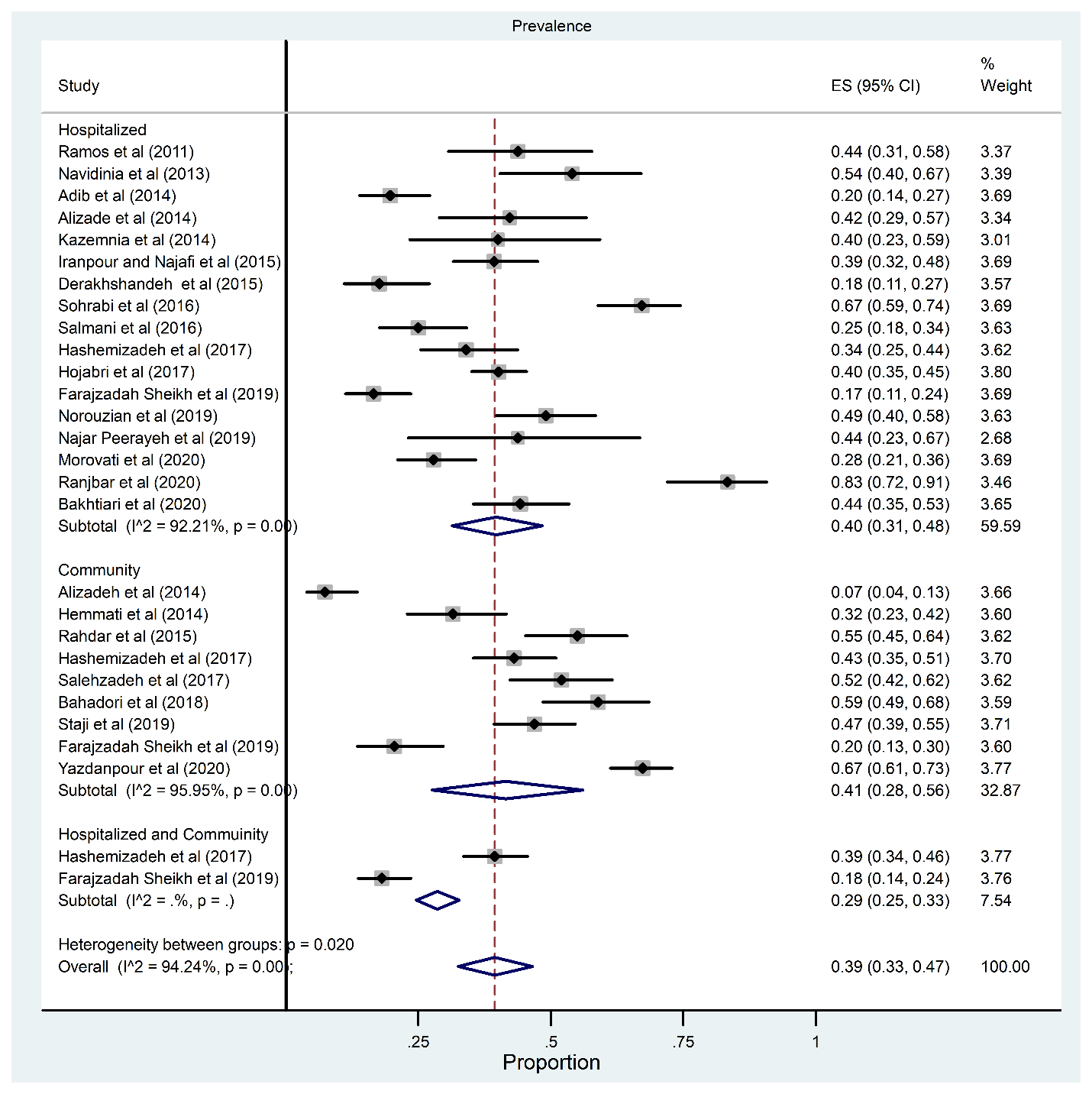


Figure 2: The subgroup analysis results based on source of patients (Phylogroup B_2_)


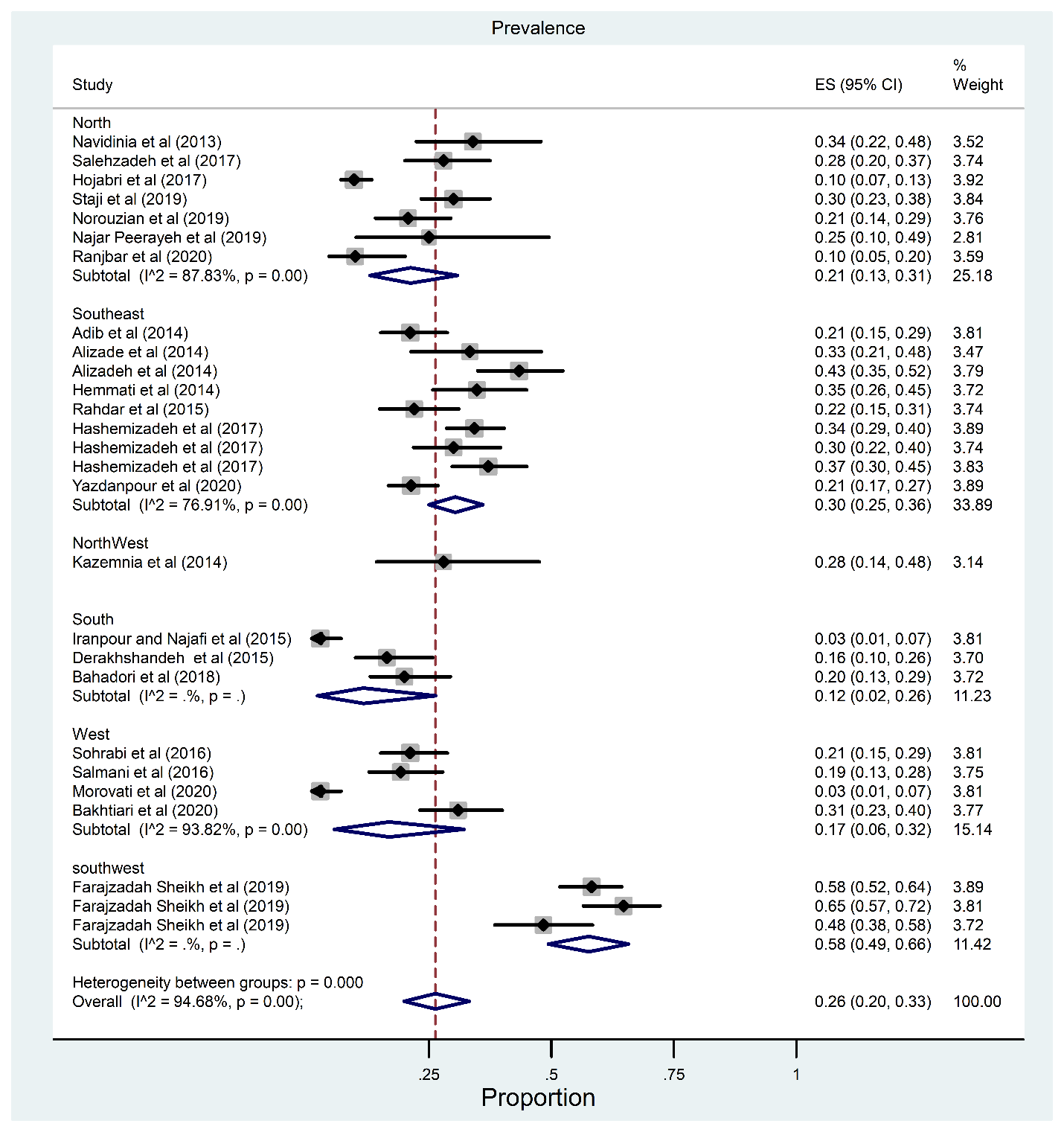


Figure 3: The subgroup analysis results based on region (Phylogroup D)


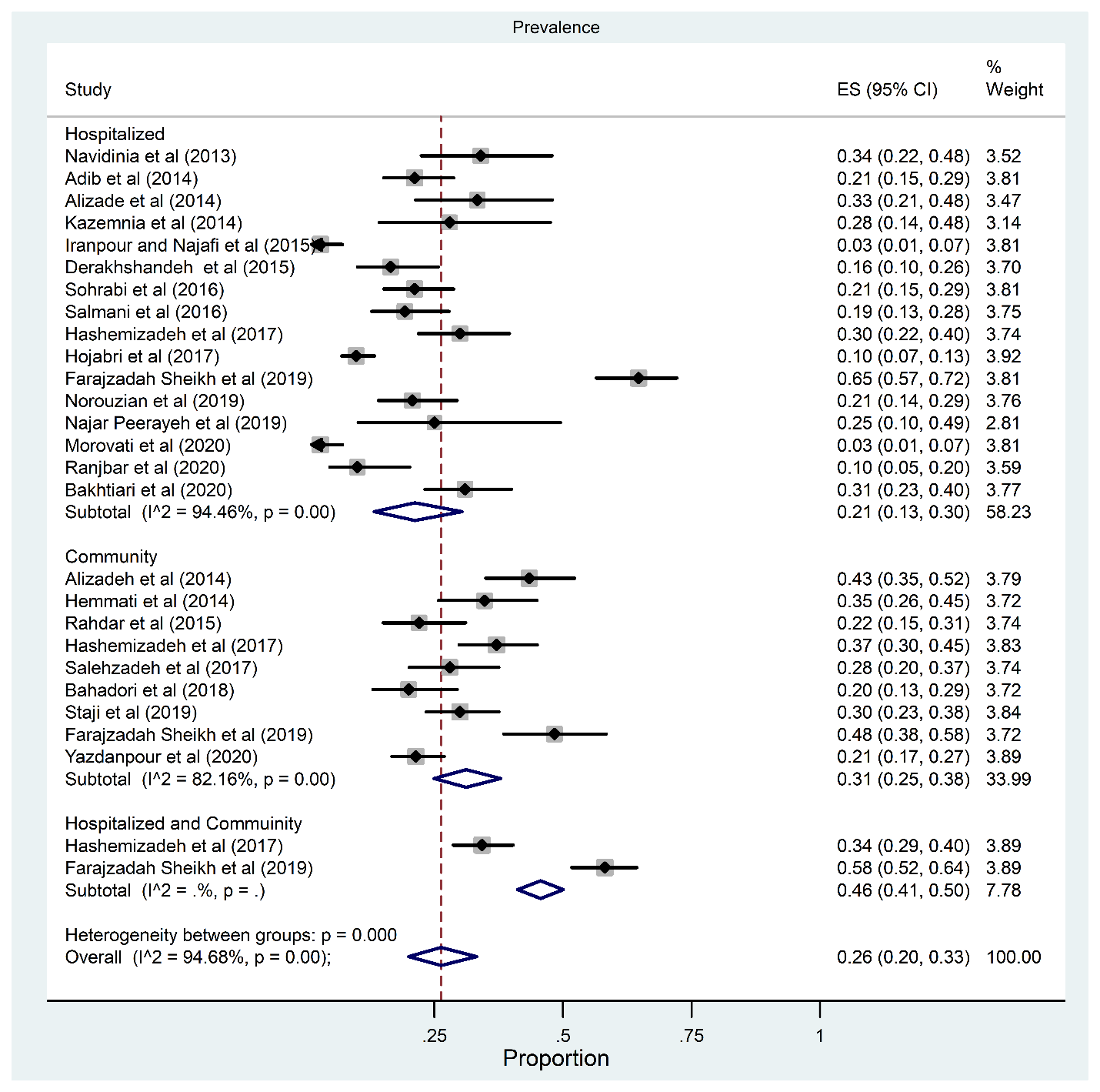


Figure 4: The subgroup analysis results based on source of patients (Phylogroup D)


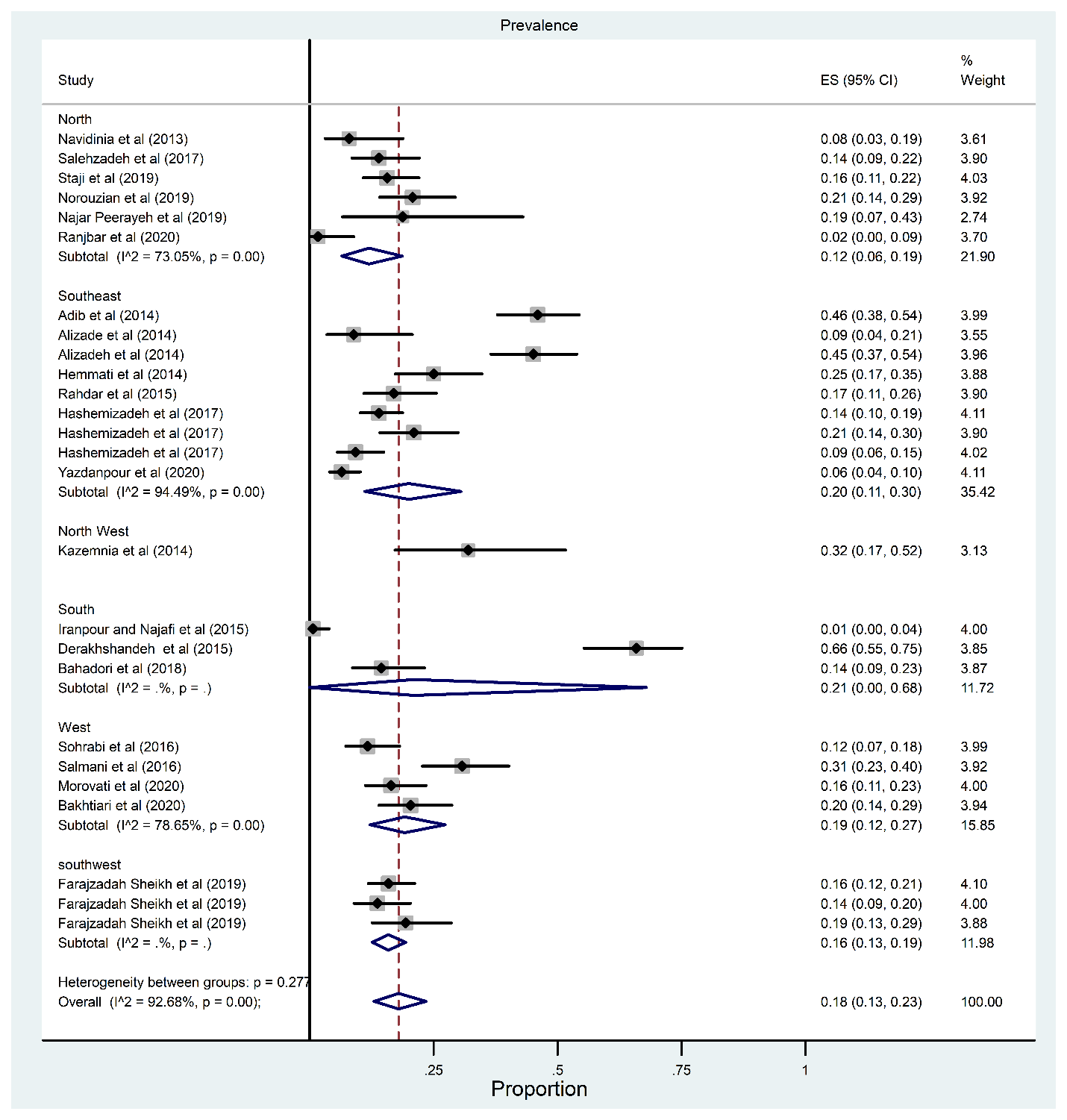


Figure 5: The subgroup analysis results based on region (Phylogroup A)


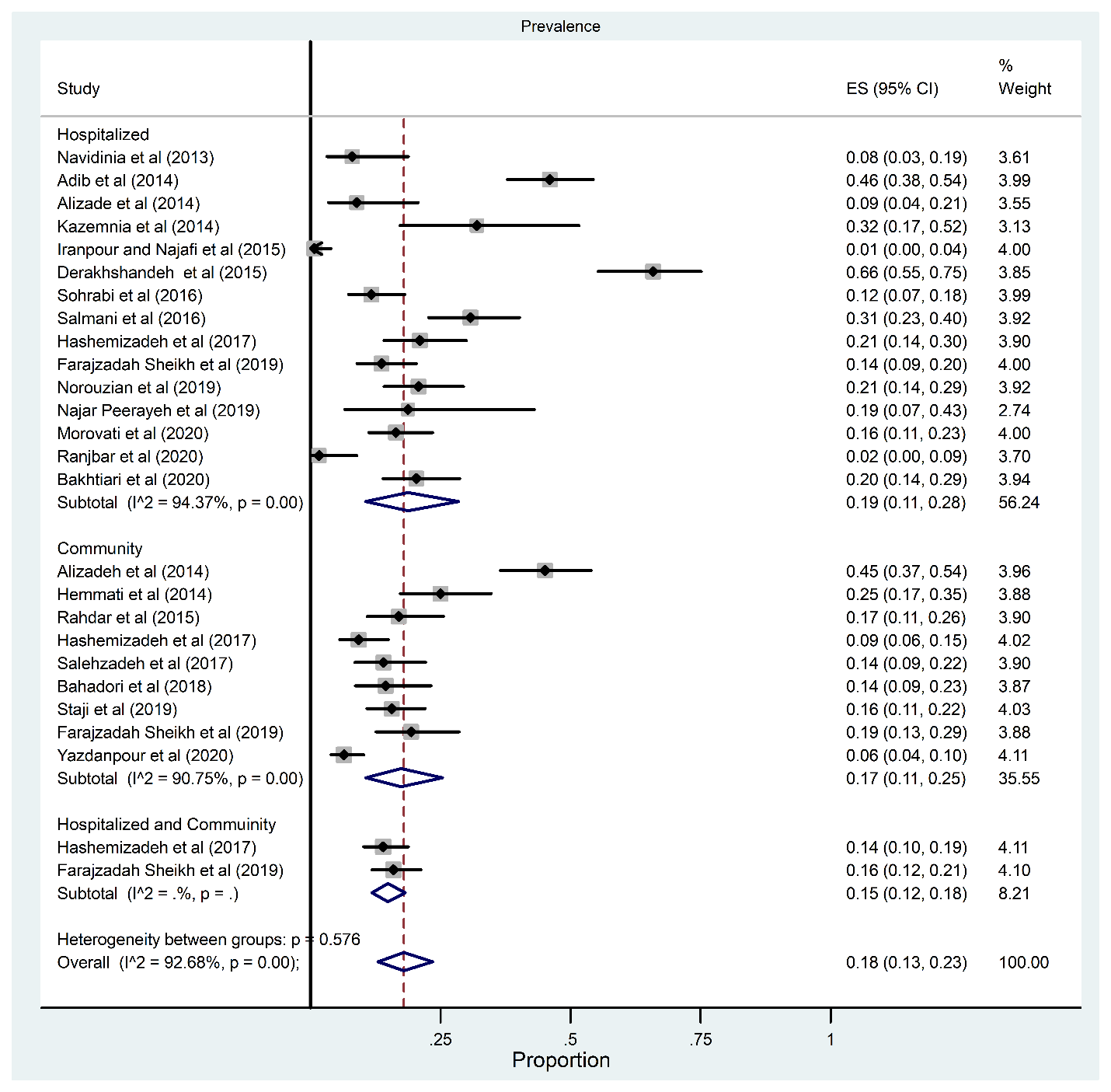


Figure 6: The subgroup analysis results based on source of patients (Phylogroup A)


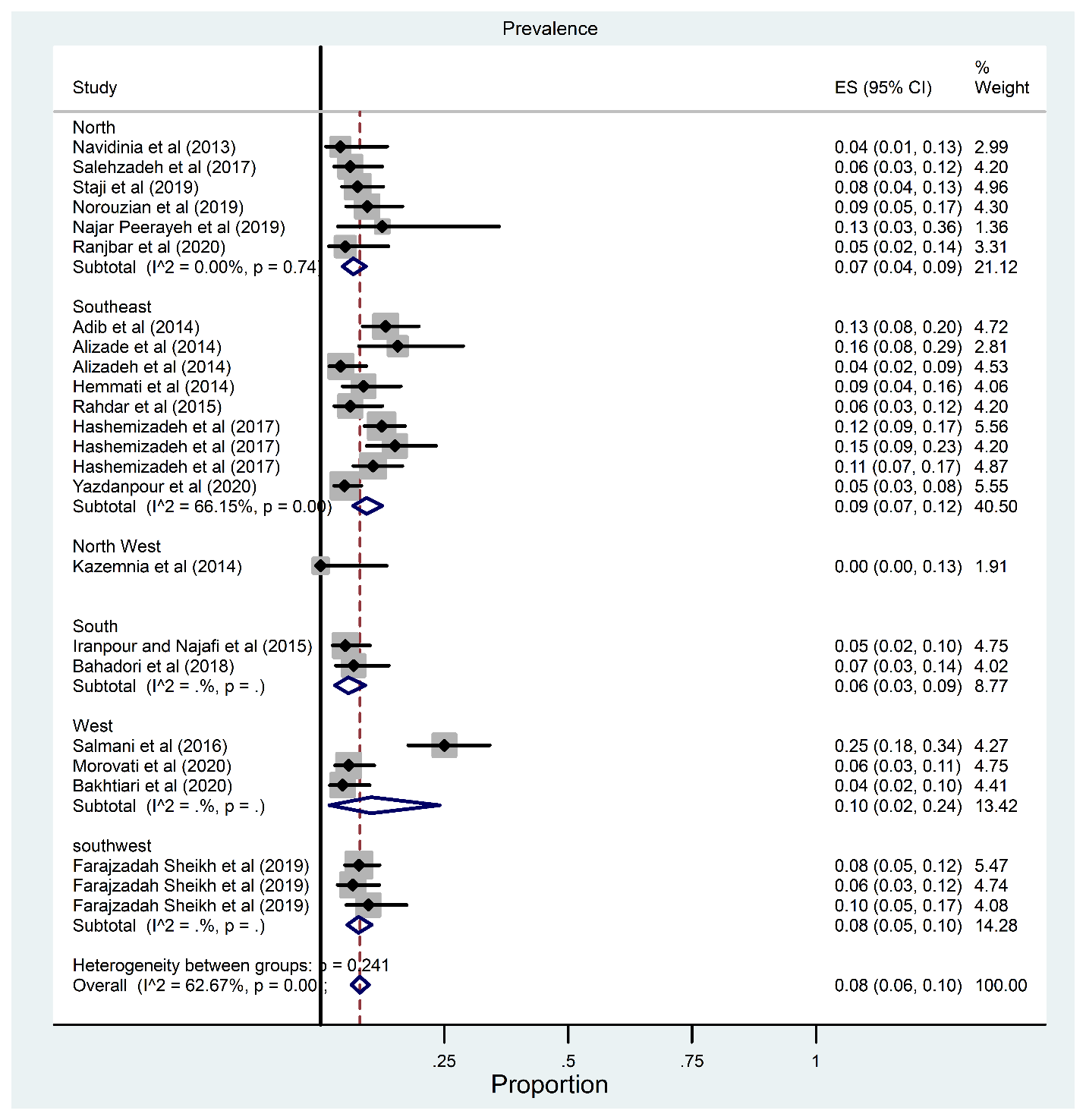


Figure 7: The subgroup analysis results based on region (Phylogroup B_1_)


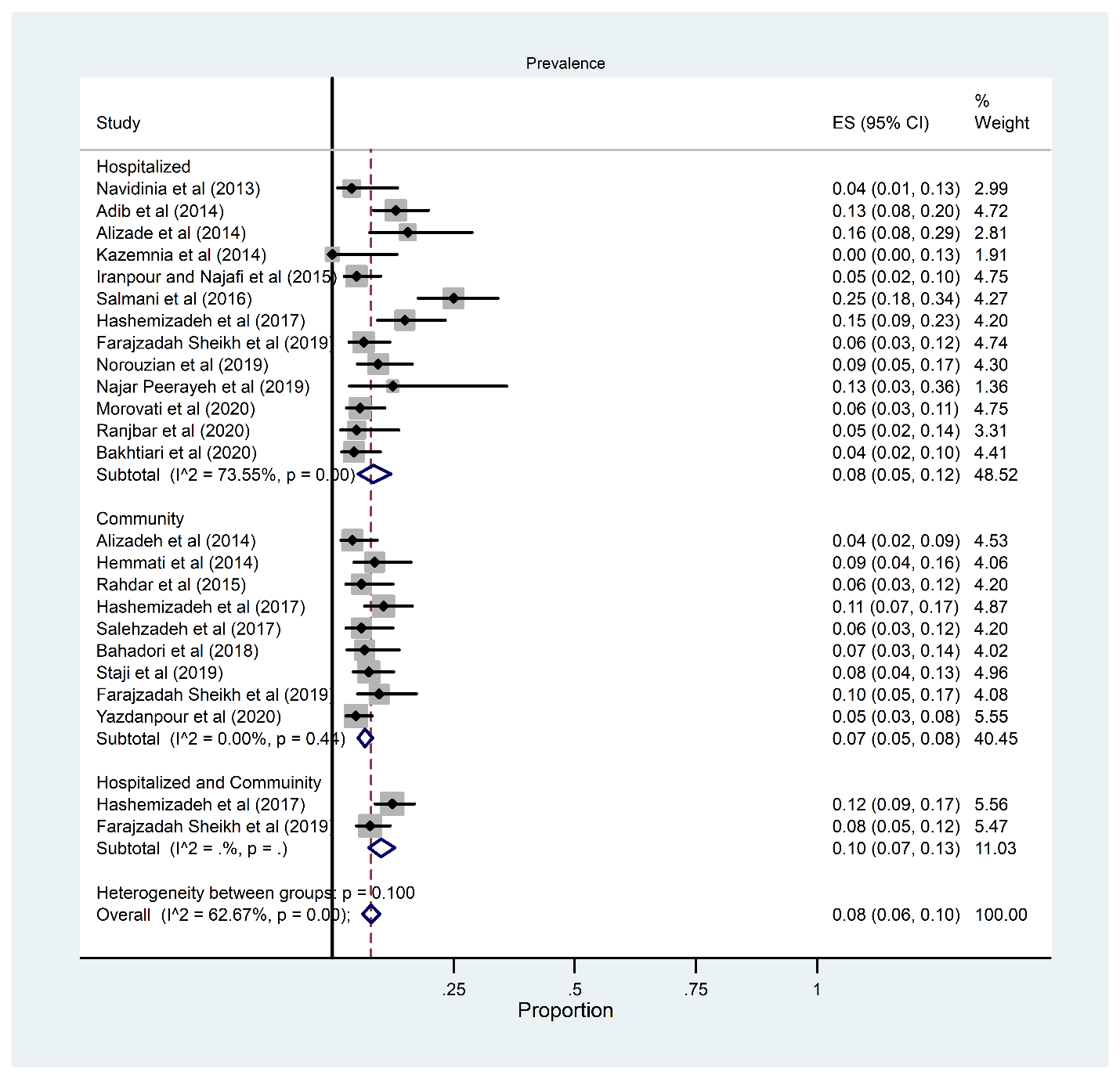


Figure 8: The subgroup analysis results based on source of patients (Phylogroup B_1_)


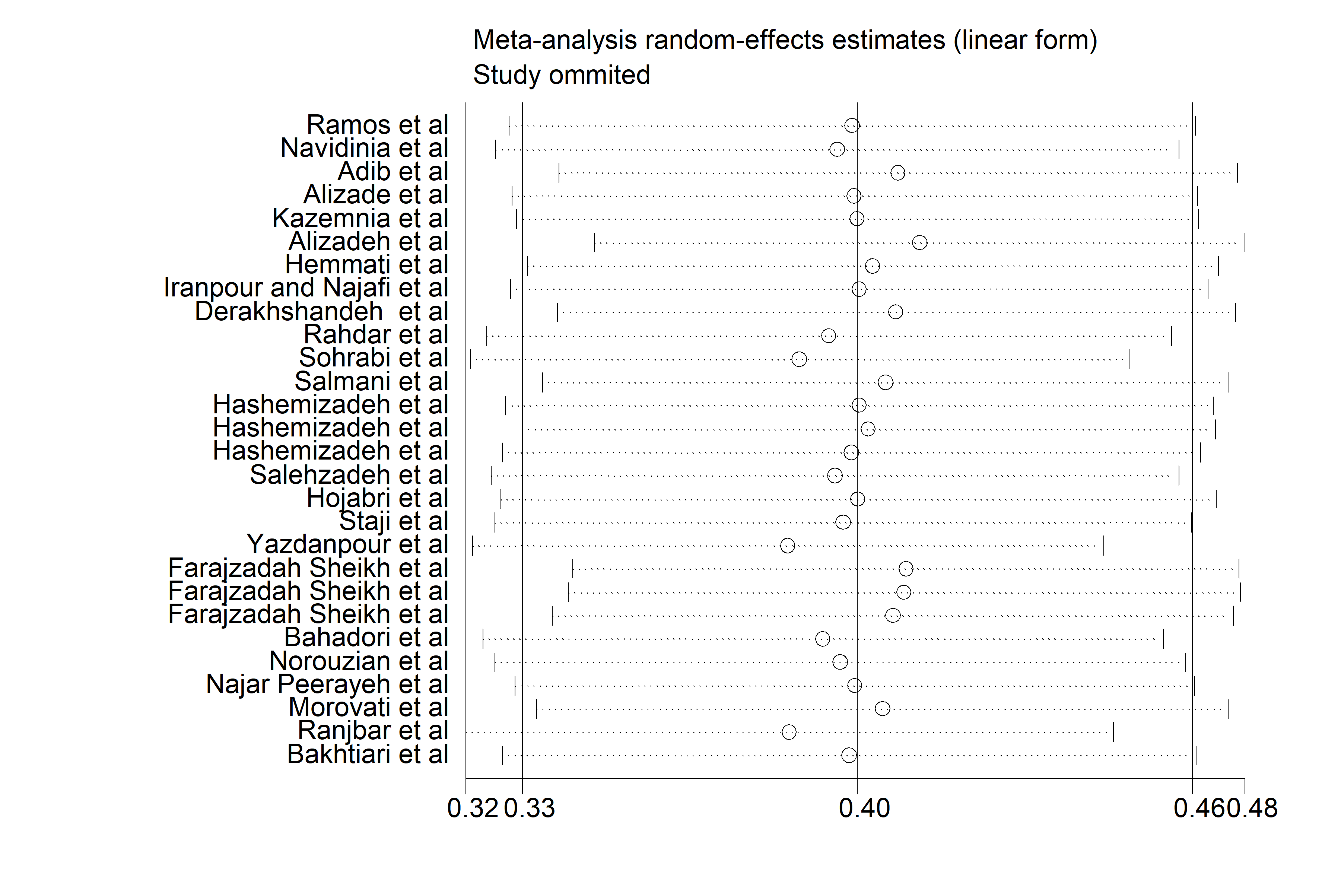


Figure 9 (A): Sensitivity analysis plot of the included studies (Phylogroup B_2_)


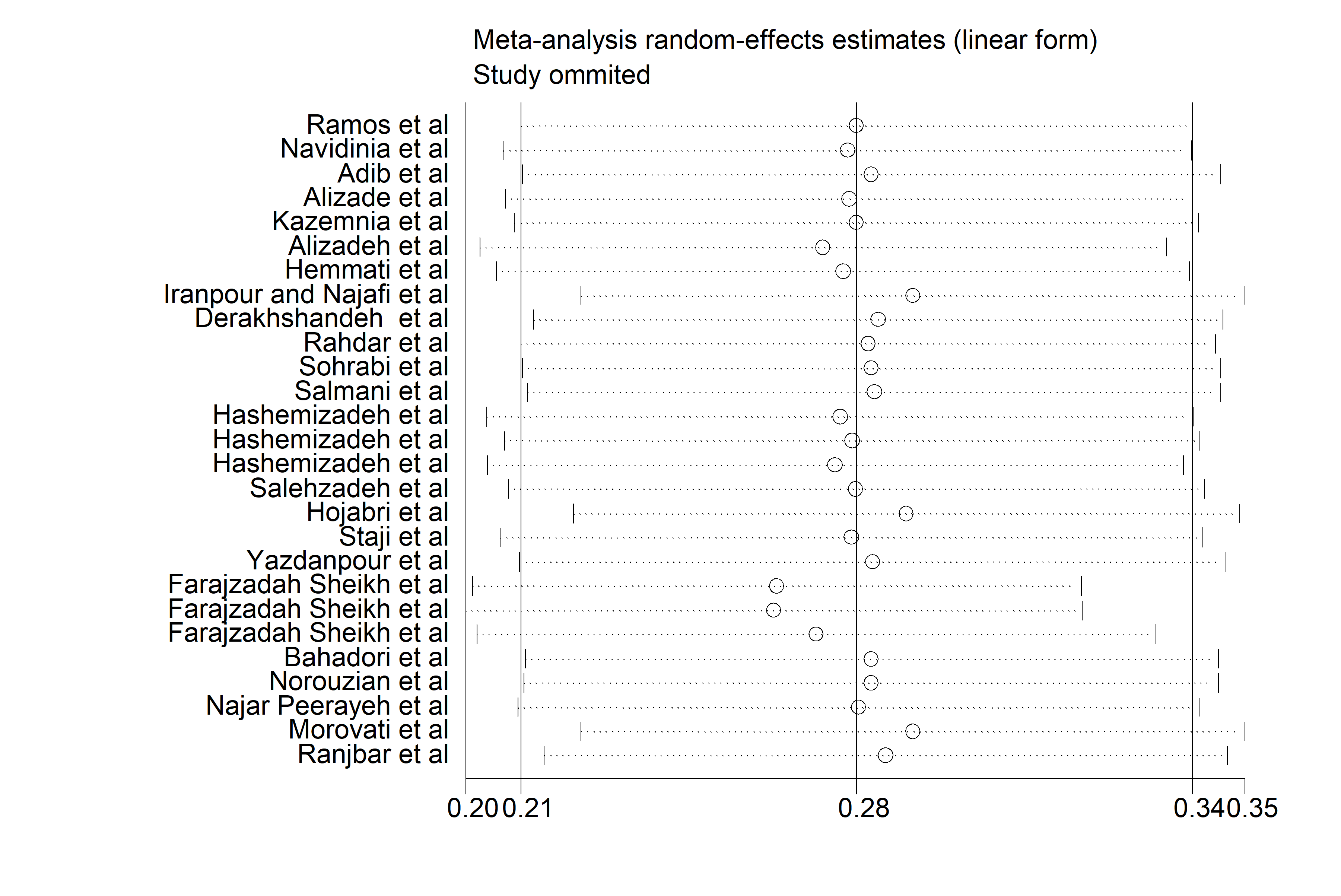


Figure 9 (B): Sensitivity analysis plot of the included studies (Phylogroup D)


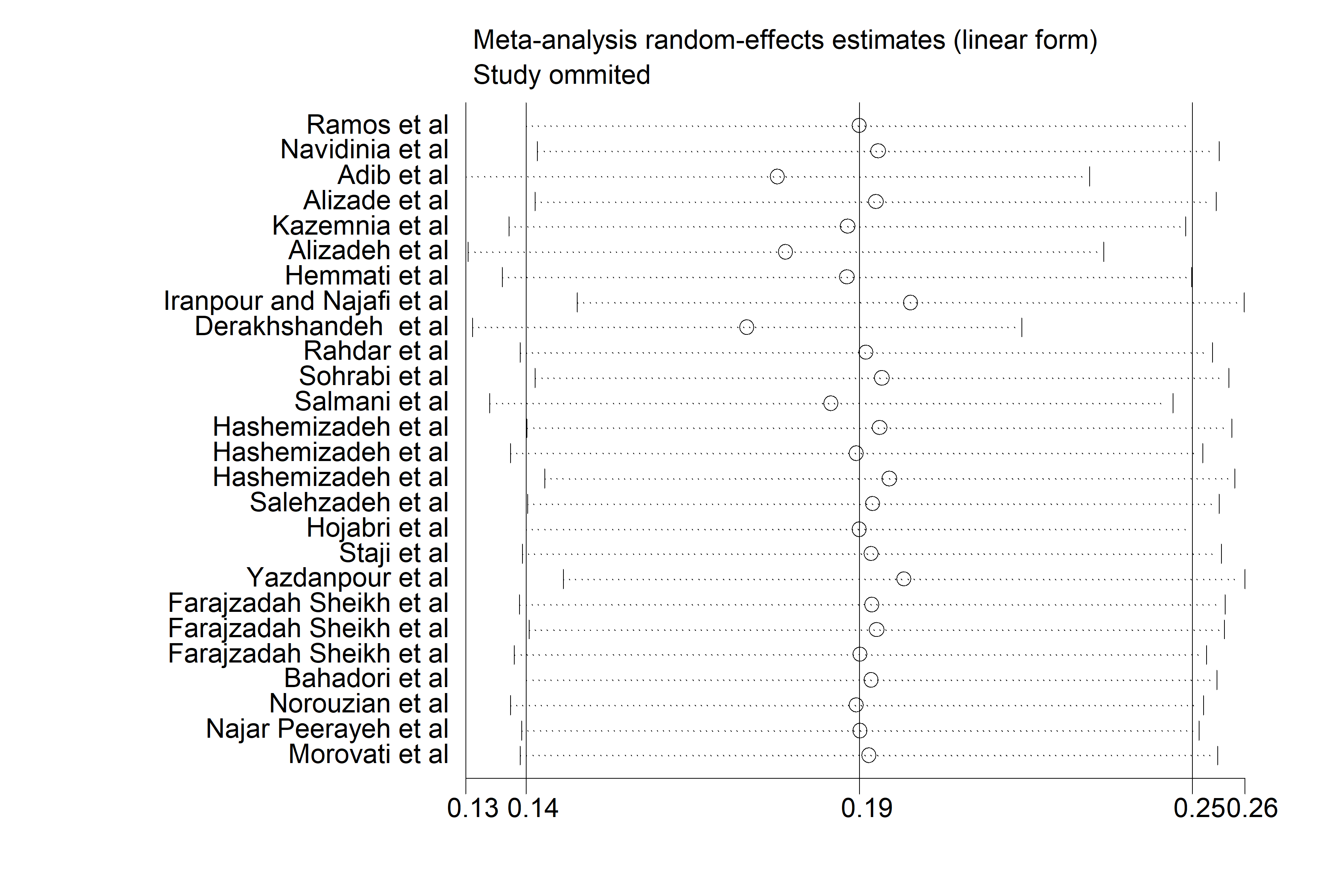


Figure 9 (C): Sensitivity analysis plot of the included studies (Phylogroup A)


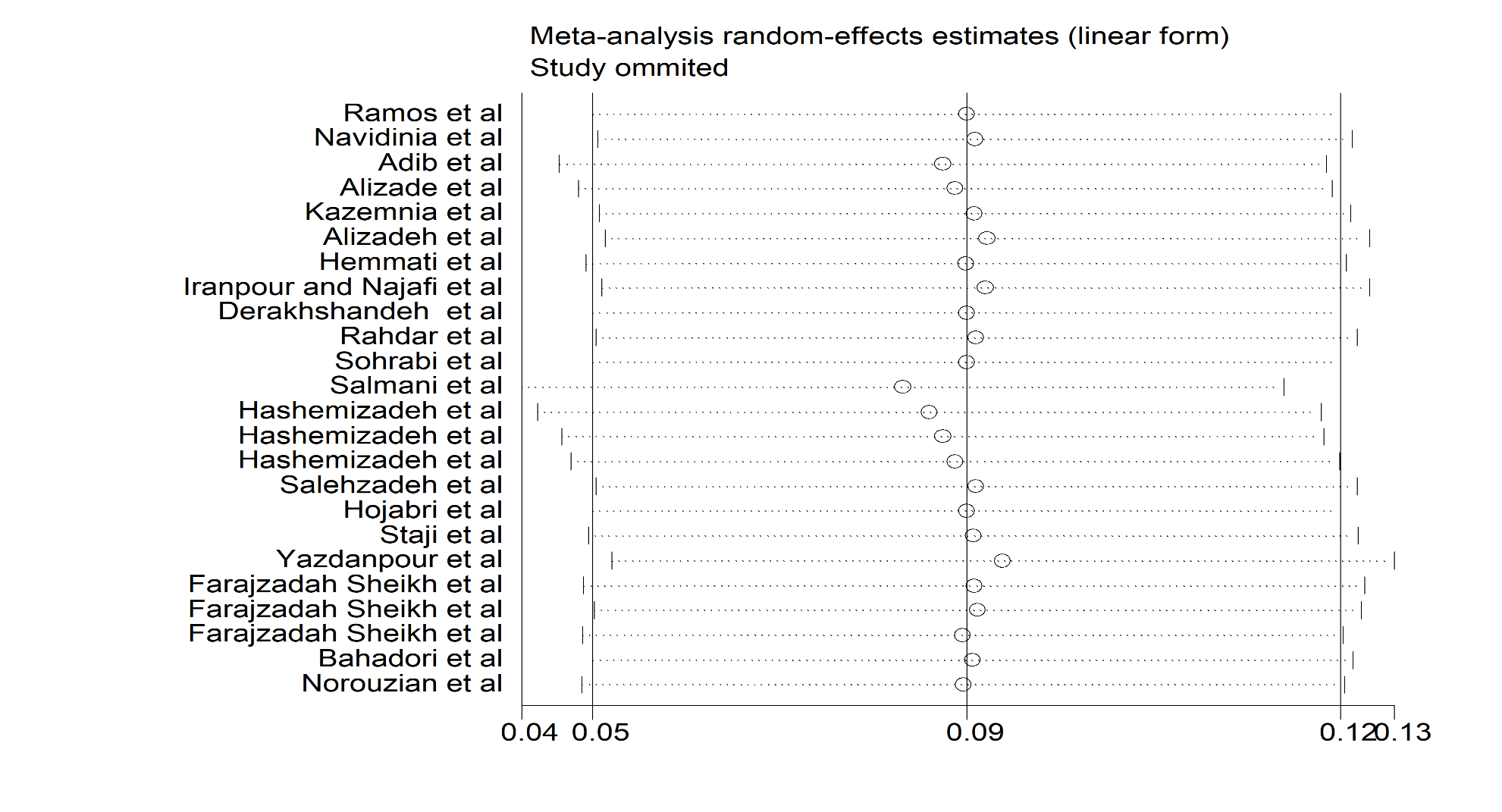


Figure 9 (D): Sensitivity analysis plot of the included studies (Phylogroup B_1_)

Table 1: Subgroup-analysis of phylogroup B2 based on regions and type of patients

| **Subgroups** | **Number of studies** | **Prevalence of resistance (95% CI)** | | | **Heterogeneity test** | | | **Publication bias tests** | |
| --- | --- | --- | --- | --- | --- | --- | --- | --- | --- |
|  |  | **Prevalence** | **Lower limit** | **Up limit** | **P-value** | **I^2^**  **(%)** | **X2** | **z** | **P-value** |
| B2 | 28 | 39% | 33 | 47 | 0.001 | 94.24 | 468.47 | 0.51 | 0.60 |
| North | 8 | 52 | 43 | 61 | 0.001 | 84.42 | 44.94 | 15.70 | 0.001 |
| Southeast | 9 | 37 | 24 | 51 | 0.001 | 95.94 | 196.98 | 8.45 | 0.001 |
| Northwest | 1 | 40 | 23 | 59 | - | - | - | 5.96 | 0.001 |
| South | 3 | 38 | 17 | 61 | - | - | - | 5.27 | 0.001 |
| west | 4 | 41 | 22 | 61 | 0.001 | 95.10 | 61.28 | 6.37 | 0.001 |
| Southwest | 3 | 18 | 15 | 22 | - | - | - | 17.21 | 0.001 |
| Heterogeneity |  |  |  |  | 0.001 | 94.24 |  |  |  |
| Hospitalized | 17 | 40 | 31 | 48 | 0.001 | 92.21 | 205.31 | 14.20 | 0.001 |
| Community | 9 | 41 | 28 | 56 | 0.001 | 95.95 | 197.76 | 8.87 | 0.001 |
| Heterogeneity |  |  |  |  | 0.02 | 94.24 |  |  |  |

Table 2: Subgroup-analysis of phylogroup D based on regions and type of patients

| **Subgroups** | **Number of studies** | **Prevalence of resistance (95% CI)** | | | **Heterogeneity test** | | | **Publication bias tests** | |
| --- | --- | --- | --- | --- | --- | --- | --- | --- | --- |
|  |  | **Prevalence** | **Lower limit** | **Up limit** | **P-value** | **I^2^**  **(%)** | **X2** | **z** | **P-value** |
| D | 27 | 26% | 20 | 33 | 0.001 | 94.68 | 488.47 | 0.1 | 0.91 |
| North | 7 | 21 | 13 | 31 | 0.001 | 87.83 | 49.32 | 7.66 | 0.001 |
| Southeast | 9 | 30 | 25 | 36 | 0.001 | 76.91 | 34.65 | 17.71 | 0.001 |
| Northwest | 1 | 24 | 14 | 48 | - | - | - | 4.73 | 0.001 |
| South | 3 | 12 | 2 | 26 | - | - | - | 3.18 | 0.001 |
| west | 4 | 17 | 6 | 32 | 0.001 | 93.82 | 48.56 | 4.21 | 0.001 |
| Southwest | 3 | 58 | 49 | 66 | - | - | - | 19.50 | 0.001 |
| Heterogeneity |  |  |  |  | 0.001 | 94.68 |  |  |  |
| Hospitalized | 16 | 21 | 13 | 30 | 0.001 | 94.46 | 270.74 | 8.05 | 0.001 |
| Community | 9 | 31 | 25 | 38 | 0.001 | 82.16 | 44.84 | 15.53 | 0.001 |
| Heterogeneity |  |  |  |  | 0.001 | 94.68 |  |  |  |

| **Subgroups** | **Number of studies** | **Prevalence of resistance (95% CI)** | | | **Heterogeneity test** | | | **Publication bias tests** | |
| --- | --- | --- | --- | --- | --- | --- | --- | --- | --- |
|  |  |  |  |  |  |  |  | Begg’s test | |
|  |  | Prevalence | Lower limit | Up limit | P-value | I^2^  (%) | X2 | z | P-value |
| A | 26 | 18% | 13 | 23 | 0.001 | 92.68 | 341.53 | 1.52 | 0.12 |
| North | 6 | 12% | 6 | 19 | 0.001 | 73.05 | 18.55 | 6.34 | 0.001 |
| Southeast | 9 | 20% | 11 | 30 | 0.001 | 94.49 | 145.2 | 6.81 | 0.001 |
| Northwest | 1 | 32% | 17 | 52 | - | - | - | 5.15 | 0.001 |
| South | 3 | 21% | 0 | 68 | - | - | - | 1.73 | 0.001 |
| west | 4 | 19% | 12 | 27 | 0.001 | 78.65 | 14.05 | 8.44 | 0.001 |
| A Southwest | 3 | 16% | 13 | 19 | - | - | - | 15.97 | 0.001 |
| **Heterogeneity** |  |  |  |  | 0.28 | 92.68 |  |  |  |
| Hospitalized | 15 | 19% | 11 | 28 | 0.001 | 94.37 | 248.48 | 6.81 | 0.001 |
| Community | 9 | 17% | 11 | 25 | 0.001 | 90.75 | 86.46 | 7.93 | 0.001 |
| **Heterogeneity** |  |  |  |  | 0.58 | 92.68 |  |  |  |

Table 3: Subgroup-analysis of phylogroup A based on regions and type of patients

Table 4: Subgroup-analysis of phylogroup B1 based on regions and type of patients

| Subgroups | Number of studies | Prevalence of resistance (95% CI) | | | Heterogeneity test | | | Publication bias tests | |
| --- | --- | --- | --- | --- | --- | --- | --- | --- | --- |
|  |  |  |  |  |  |  |  | Begg’s test | |
|  |  | Prevalence | Lower limit | Up limit | P-value | I^2^  (%) | X2 | z | P-value |
| B1 | 24 | 8% | 6 | 10 | 0.001 | 62.67 | 61.62 | 0.07 | 0.94 |
| North | 6 | 7% | 4 | 9 | 0.74 | 0 | 2.71 | 9.15 | 0.001 |
| Southeast | 9 | 9% | 7 | 12 | 0 | 66.15 | 23.63 | 10.67 | 0.001 |
| Northwest | 1 | 0 | 0 | 13 | - | - | - | 0 | 1 |
| South | 2 | 6% | 3 | 9 | - | - | - | 6.07 | 0.001 |
| West | 3 | 10% | 2 | 24 | - | - | - | 3.03 | 0.001 |
| Southwest | 3 | 8% | 5 | 10 | - | - | - | 10.51 | 0.001 |
| Heterogeneity |  |  |  |  | 0.24 | 62.67 |  |  |  |
| Hospitalized | 13 | 8% | 5 | 12 | 0.001 | 73.55 | 45.36 | 8.17 | 0.001 |
| Community | 9 | 7% | 5 | 8 | 0.44 | 0 | 7.97 | 15.22 | 0.001 |
| Heterogeneity |  | 0 |  |  | 0.10 | 62.67 |  |  |  |

Table 5: Subgroups meta-analysis of antibiotic resistance prevalence in phylogroup B_2_

| Subgroups | Number of studies | Prevalence of resistance (95% CI) | | | Heterogeneity test | | Publication bias tests | | | |
| --- | --- | --- | --- | --- | --- | --- | --- | --- | --- | --- |
|  |  |  |  |  |  |  | Begg’s test | | Egger’s test | |
|  |  | Prevalence | Lower limit | Up limt | P-value | I^2^  (%) | z | P-value | t | P-value |
| AMP | 6 | 76% | 58 | 90 | <0.001 | 89.57 | 0.57 | 0.56 | -0.10 | 0.92 |
| CFM | 3 | 70% | 34 | 96 | <0.001 | 90.84 | - | - | - | - |
| NAL | 6 | 60% | 33 | 84 | <0.001 | 94.25 | 0.19 | 0.84 | 0.5 | 0.64 |
| CTX | 7 | 51% | 46 | 57 | 0.09 | 44.5 | 0.46 | 0.64 | 0.69 | 0.52 |
| CIP | 7 | 46% | 33 | 59 | <0.001 | 80.70 | 0 | 1 | 0.16 | 0.87 |
| SXT | 7 | 44% | 26 | 63 | <0.001 | 90.56 | 0 | 1 | -0.30 | 0.77 |
| GEN | 8 | 25% | 16 | 35 | <0.001 | 76.22 | 0.99 | 0.32 | 0.17 | 0.86 |
| AMK | 5 | 8% | 2 | 17 | <0.001 | 81.33 | 0.24 | 0.8 | 0.21 | 0.84 |
| IMP | 6 | 7% | 0 | 21 | <0.001 | 92.52 | 0 | 1 | 0.26 | 0.8 |
| NIT | 5 | 2% | 0 | 5 | 0.18 | 35.65 | 0.24 | 0.8 | -0.26 | 0.81 |

GEN: gentamicin; SXT: trimethoprim/sulfamethoxazole; NAL: nalidixic acid; CIP: ciprofloxacin; IMP: imipenem; CFM: cefixime; CTX: cefotaxime; AMK: amikacin; NIT: nitrofurantoin; AMP: ampicillin; CEF: ceftriaxone; CAZ: ceftazidime

Table 6: Subgroups meta-analysis of antibiotic resistance prevalence in phylogroup D

| **Subgroups** | **Number of studies** | **Prevalence of resistance (95% CI)** | | | **Heterogeneity test** | | **Publication bias tests** | | | |
| --- | --- | --- | --- | --- | --- | --- | --- | --- | --- | --- |
|  |  |  |  |  |  |  | Begg’s test | | Egger’s test | |
|  |  | **Prevalence** | **Lower limit** | **Up limt** | **P-value** | **I^2^**  **(%)** | z | P-value | t | P-value |
| CFM | 3 | 80% | 53 | 98 | 0.16 | 45.54 | 1.04 | 0.29 | -5.79 | 0.1 |
| AMP | 6 | 64% | 28 | 94 | <0.001 | 93.87 | 0.38 | 0.7 | 1.83 | 0.14 |
| NAL | 6 | 46% | 4 | 91 | <0.001 | 95.52 | 0.57 | 0.56 | 1.57 | 0.19 |
| CTX | 7 | 46% | 29 | 63 | 0.02 | 60.49 | 0.46 | 0.64 | -0.47 | 0.66 |
| SXT | 7 | 44% | 16 | 74 | <0.001 | 93.40 | 0 | 1 | 1.52 | 0.19 |
| CIP | 7 | 37% | 13 | 64 | <0.001 | 90.89 | 0.46 | 0.64 | 0.86 | 0.43 |
| IMP | 7 | 37% | 13 | 64 | <0.001 | 90.89 | 0.46 | 0.64 | 0.86 | 0.43 |
| CAZ | 4 | 22% | 5 | 45 | 0.01 | 73.72 | -0.34 | 1 | 0.8 | 0.5 |
| GEN | 8 | 21% | 4 | 44 | <0.001 | 88.32 | 0.5 | 0.61 | 1.1 | 0.31 |
| CEF | 4 | 17% | 0 | 54 | <0.001 | 78.02 | 0 | 1 | 1.13 | 0.37 |
| AMK | 5 | 5% | 0 | 15 | 0.04 | 60.68 | 0 | 1 | -0.12 | 0.91 |
| NIT | 5 | 0% | 0 | 3 | 0 | 0 | 0.98 | 0.32 | -1.99 | 0.14 |

GEN: gentamicin; SXT: trimethoprim/sulfamethoxazole; NAL: nalidixic acid; CIP: ciprofloxacin; IMP: imipenem; CFM: cefixime; CTX: cefotaxime; AMK: amikacin; NIT: nitrofurantoin; AMP: ampicillin; CEF: ceftriaxone; CAZ: ceftazidime

Table 7: Subgroups meta-analysis of antibiotic resistance prevalence in phylogroup A

| **Subgroups** | **Number of studies** | **Prevalence of resistance (95% CI)** | | | **Heterogeneity test** | | **Publication bias tests** | | | |
| --- | --- | --- | --- | --- | --- | --- | --- | --- | --- | --- |
|  |  |  |  |  |  |  | Begg’s test | | Egger’s test | |
|  |  | **Prevalence** | **Lower limit** | **Up limt** | **P-value** | **I^2^**  **(%)** | z | P-value | t | P-value |
| AMP | 6 | 59% | 27 | 89 | <0.001 | 75.65 | 0.19 | 0.85 | 1.47 | 0.21 |
| NAL | 6 | 58% | 6 | 100 | <0.001 | 93.45 | 0.38 | 0.7 | 1.38 | 0.24 |
| CTX | 7 | 57% | 46 | 67 | 0 | 0 | 0 | 1 | -0.96 | 0.37 |
| CFM | 3 | 54% | 38 | 69 | 0 | 0 | - | - | - | - |
| SXT | 7 | 43% | 14 | 75 | <0.001 | 93.64 | 0.76 | 0.44 | 0.76 | 0.48 |
| CIP | 7 | 42% | 25 | 61 | 0.01 | 62.75 | 1.67 | 0.09 | 1.91 | 0.11 |
| CAZ | 4 | 25% | 3 | 55 | 0.05 | 61.48 | - | - | - | - |
| CEF | 4 | 21% | 5 | 42 | 0.3 | 19.03 | - | - | - | - |
| GEN | 8 | 13% | 1 | 32 | <0.001 | 67.54 | 1.36 | 0.17 | 0.66 | 0.53 |
| AMK | 5 | 3% | 0 | 16 | 0.05 | 58.77 | 0 | 1 | -0.66 | 0.55 |
| IMP | 6 | 1% | 0 | 12 | 0.02 | 61.83 | 1.69 | 0.09 | 3.69 | 0.02 |
| NIT | 5 | 0% | 0 | 2 | 0 | 0 | 1.01 | 0.31 | 0.48 | 0.66 |

GEN: gentamicin; SXT: trimethoprim/sulfamethoxazole; NAL: nalidixic acid; CIP: ciprofloxacin; IMP: imipenem; CFM: cefixime; CTX: cefotaxime; AMK: amikacin; NIT: nitrofurantoin; AMP: ampicillin; CEF: ceftriaxone; CAZ: ceftazidime

Table 8: Subgroups meta-analysis of antibiotic resistance prevalence in phylogroup B_1_

| **Subgroups** | **Number of studies** | **Prevalence of resistance (95% CI)** | | | **Heterogeneity test** | | **Publication bias tests** | | | |
| --- | --- | --- | --- | --- | --- | --- | --- | --- | --- | --- |
|  |  |  |  |  |  |  | Begg’s test | | Egger’s test | |
|  |  | **Prevalence** | **Lower limit** | **Up limt** | **P-value** | **I^2^**  **(%)** | z | P-value | t | P-value |
| AMP | 6 | 32% | 0 | 90 | 0.4 | 3.01 | -0.19 | 1 | 0.23 | 0.83 |
| CIP | 5 | 30% | 16 | 46 | 0 | 0 | 1.22 | 0.22 | 1.40 | 0.25 |
| CEF | 4 | 4% | 0 | 16 | 0.38 | 2.92 | - | - | - | - |
| SXT | 6 | 3% | 0 | 76 | <0.001 | 76.99 | 0.57 | 0.56 | 0.57 | 0.6 |
| CFM | 3 | 3% | 0 | 100 | <0.001 | 84.17 | - | - | - | - |
| GEN | 6 | 2% | 0 | 10 | 0.23 | 27.07 | 2.63 | 0.009 | 2.94 | 0.04 |
| NAL | 6 | 1% | 0 | 56 | 0.05 | 53.96 | 0.75 | 0.45 | 0.76 | 0.49 |
| IMP | 6 | 0% | 0 | 0 | 0 | 0 | 0.24 | 0.8 | -5.19 | 0.01 |
| CTX | 6 | 0% | 0 | 16 | 0.33 | 13.72 | 0.38 | 0.7 | 0.08 | 0.94 |
| AMK | 5 | 0% | 0 | 0 | 0 | 0 | 0.24 | 0.8 | -1.23 | 0.3 |
| NIT | 5 | 0% | 0 | 6 | 0 | 0 | 0.51 | 0.61 | -0.99 | 0.39 |
| CAZ | 4 | 0% | 0 | 12 | 0.29 | 20.34 | - | - | - | - |

GEN: gentamicin; SXT: trimethoprim/sulfamethoxazole; NAL: nalidixic acid; CIP: ciprofloxacin; IMP: imipenem; CFM: cefixime; CTX: cefotaxime; AMK: amikacin; NIT: nitrofurantoin; AMP: ampicillin; CEF: ceftriaxone; CAZ: ceftazidime

Table 9: Subgroups meta-analysis of virulence factor prevalence in phylogroup B_2_

| **Subgroups** | **Number of studies** | **Prevalence of resistance (95% CI)** | | | **Heterogeneity test** | | **Publication bias tests** | | | |
| --- | --- | --- | --- | --- | --- | --- | --- | --- | --- | --- |
|  |  |  |  |  |  |  | Begg’s test | | Egger’s test | |
|  |  | **Prevalence** | **Lower limit** | **Up limt** | **P-value** | **I^2^**  **(%)** | z | P-value | t | P-value |
| *fimH* | 5 | 90% | 82 | 95 | 0.01 | 68.20 | 1.22 | 0.22 | 3.91 | 0.03 |
| *papC* | 3 | 81% | 55 | 98 | <0.001 | 94.68 | 0 | 1 | -0.41 | 0.75 |
| *iucD* | 3 | 43% | 22 | 64 | 0.01 | 78.20 | 1.04 | 0.29 | -3.15 | 0.19 |
| *sfa* | 9 | 34% | 15 | 57 | <0.001 | 95.69 | 0.21 | 0.83 | 0.27 | 0.79 |
| *papEF* | 3 | 31% | 7 | 61 | 0.01 | 77.74 | 0 | 1 | -0.39 | 0.76 |
| *hlya* | 4 | 23% | 6 | 46 | <0.001 | 93.22 | 1.02 | 0.30 | -2.32 | 0.14 |
| *cnfi* | 5 | 22% | 9 | 38 | <0.001 | 86.84 | 0.73 | 0.46 | -1.20 | 0.31 |
| *hly* | 4 | 21% | 12 | 31 | 0.04 | 62.88 | 0.34 | 0.73 | -2.04 | 0.17 |
| *afa* | 4 | 7% | 3 | 12 | 0.77 | 0 | 0 | 1 | -0.87 | 0.47 |

Table 10: Subgroups meta-analysis of virulence factor prevalence in phylogroup D

| **Subgroups** | **Number of studies** | **Prevalence of resistance (95% CI)** | | | **Heterogeneity test** | | **Publication bias tests** | | | |
| --- | --- | --- | --- | --- | --- | --- | --- | --- | --- | --- |
|  |  |  |  |  |  |  | Begg’s test | | Egger’s test | |
|  |  | **Prevalence** | **Lower limit** | **Up limt** | **P-value** | **I^2^**  **(%)** | z | P-value | t | P-value |
| *fimH* | 5 | 80% | 58 | 96 | <0.001 | 91.03 | 0.24 | 0.80 | -1.45 | 0.24 |
| *papC* | 3 | 79% | 57 | 95 | 0.06 | 64.77 | 0 | 1 | -2.26 | 0.26 |
| *iucD* | 3 | 43% | 33 | 54 | 0.41 | 0 | 0 | 1 | -0.82 | 0.56 |
| *afa* | 4 | 18% | 10 | 27 | 0.89 | 0 | -0.34 | 1 | 0.25 | 0.82 |
| *sfa* | 9 | 18% | 6 | 33 | <0.001 | 88.90 | 0.84 | 0.40 | 1.00 | 0.34 |
| *hlya* | 4 | 17% | 0 | 52 | <0.001 | 95.72 | -0.34 | 1 | 0.05 | 0.96 |
| *cnfi* | 5 | 15% | 4 | 30 | <0.001 | 77.16 | 1.22 | 0.22 | -3.18 | 0.05 |
| *papEF* | 3 | 11% | 5 | 18 | 0.76 | 0 | 0 | 1 | 1.36 | 0.40 |
| *hly* | 4 | 10% | 0 | 30 | <0.001 | 88.93 | -0.34 | 1 | -0.54 | 0.64 |

| **Subgroups** | **Number of studies** | **Prevalence of resistance (95% CI)** | | | **Heterogeneity test** | | **Publication bias tests** | | | |
| --- | --- | --- | --- | --- | --- | --- | --- | --- | --- | --- |
|  |  |  |  |  |  |  | Begg’s test | | Egger’s test | |
|  |  | **Prevalence** | **Lower limit** | **Up limt** | **P-value** | **I^2^**  **(%)** | z | P-value | t | P-value |
| *fimH* | 5 | 87% | 64 | 100 | <0.001 | 75.32 | -0.24 | 1 | -0.37 | 0.73 |
| *papC* | 3 | 75% | 10 | 100 | <0.001 | 85.72 | 0 | 1 | -0.65 | 0.63 |
| *iucD* | 3 | 39% | 19 | 60 | 0.08 | 60.17 | 1.04 | 0.29 | -4.70 | 0.13 |
| *sfa* | 9 | 10% | 0 | 31 | <0.001 | 89.51 | 0.52 | 0.60 | 0.24 | 0.81 |
| *papEF* | 3 | 7% | 2 | 14 | 0.57 | 0 | 0 | 1 | 7.16 | 0.08 |
| *hlya* | 4 | 4% | 0 | 31 | <0.001 | 81.20 | -0.34 | 1 | -0.12 | 0.91 |
| *hly* | 4 | 4% | 0 | 24 | <0.001 | 81.07 | 1.02 | 0.30 | 0.54 | 0.64 |
| *cnfi* | 5 | 1% | 0 | 6 | 0.57 | 0 | 0.24 | 0.80 | 0.32 | 0.77 |
| *afa* | 4 | 1% | 0 | 7 | 0.50 | 0 | 0.34 | 0.73 | 0.86 | 0.47 |

Table 11: Subgroups meta-analysis of virulence factor prevalence in phylogroup A

Table 12: Subgroups meta-analysis of virulence factor prevalence in phylogroup B_1_

| **Subgroups** | **Number of studies** | **Prevalence of resistance (95% CI)** | | | **Heterogeneity test** | | **Publication bias tests** | | | |
| --- | --- | --- | --- | --- | --- | --- | --- | --- | --- | --- |
|  |  |  |  |  |  |  | Begg’s test | | Egger’s test | |
|  |  | **Prevalence** | **Lower limit** | **Up limt** | **P-value** | **I^2^**  **(%)** | z | P-value | t | P-value |
| *fimH* | 5 | 79% | 56 | 96 | 0.01 | 71.10 | 0 | 1 | 0.57 | 0.60 |
| *papC* | 3 | 36% | 0 | 84 | <0.001 | 81.48 | 0 | 1 | -0.32 | 0.80 |
| *iucD* | 3 | 35% | 19 | 52 | 0.89 | 0 | 1.04 | 0.29 | 2.01 | 0.29 |
| *sfa* | 9 | 15% | 2 | 35 | <0.001 | 72.40 | 0.75 | 0.45 | 0.70 | 0.50 |
| *cnfi* | 5 | 9% | 1 | 23 | 0.16 | 39.58 | 0.24 | 0.80 | 0.32 | 0.77 |
| *hly* | 4 | 6% | 0 | 19 | 0.22 | 31.28 | 0.72 | 0.47 | 1.60 | 0.25 |
| *hlya* | 4 | 5% | 0 | 28 | <0.001 | 78.43 | -0.34 | 1 | -1.60 | 0.25 |
| *papEF* | 3 | 4% | 0 | 17 | 0.62 | 0 | 0 | 1 | -4.02 | 0.15 |
| *afa* | 4 | 0% | 0 | 9 | 1 | 0 | -0.25 | 1 | - | - |
